# Maternal body mass index in early pregnancy and offspring mortality up to early adulthood: A nationwide cohort study

**DOI:** 10.1371/journal.pmed.1005217

**Published:** 2026-09-01

**Authors:** Hui Wang, Hua Chen, Jette Möller, Yajun Liang, Krisztina D. László

**Affiliations:** 1 MOE-Shanghai Key Laboratory of Children’s Environmental Health, Xinhua Hospital, Shanghai Jiao Tong University School of Medicine, Shanghai, China; 2 Department of Global Public Health, Karolinska Institutet, Stockholm, Sweden; 3 Department of Maternal and Child Health, School of Public Health, Shanghai Jiao Tong University School of Medicine, Shanghai, China; 4 Department of Public Health and Caring Sciences, Uppsala University, Uppsala, Sweden; London School of Hygiene and Tropical Medicine, UNITED KINGDOM OF GREAT BRITAIN AND NORTHERN IRELAND

## Abstract

**Background:**

Obesity is increasingly common among women of childbearing age worldwide. Maternal obesity during pregnancy may have long-term implications for offspring health, but its association with offspring mortality remains not well understood. In this nationwide cohort study, we examined the associations of maternal body mass index (BMI) in early pregnancy with all-cause and cause-specific mortality in offspring.

**Methods and findings:**

We included 2,606,684 live births in Sweden during 1982–2014 (except for 1990 and 1991) and followed them from birth until death, emigration from Sweden, or December 31, 2023, whichever came first. Information on maternal BMI during early pregnancy was obtained from the Medical Birth Register and was categorized as: underweight (BMI <18.5 kg/m^2^), normal weight (18.5–24.9 kg/m^2^), overweight (25.0–29.9 kg/m^2^), obesity grade I (30.0–34.9 kg/m^2^), obesity grade II (35.0–39.9 kg/m^2^), and obesity grade III (≥40.0 kg/m^2^). Data on all-cause and cause-specific mortality in offsprings were obtained from the Cause of Death Register. Cox proportional hazard models were performed to estimate hazard ratios (HRs) and 95% confidence intervals (95% CI) for offspring mortality according to maternal BMI (both as a continuous and as a categorical variable), adjusting for maternal characteristics (age, education, and income at the childbirth, marital status, country of birth, smoking status in early pregnancy, psychiatric disorders and cardiovascular diseases before the childbirth). We used cousin comparison analysis to explore the potential influence of familial confounding factors. During a median follow-up of 22.4 years (interquartile range 14.9–30.7 years), 21,981 (0.8%) offspring died. Maternal BMI was positively associated with all-cause mortality in offspring: HR 1.03 (95% CI [1.02, 1.03]; *p* < 0.001) per 1 unit increase in BMI. Offspring of mothers with overweight or obesity (regardless of grade) during early pregnancy had higher risks of all-cause mortality than their counterparts with normal maternal BMI at the reference category; the corresponding HRs were HR 1.11 (95% CI [1.07, 1.15]; *p* < 0.001) for overweight, HR 1.27 (95% CI [1.20, 1.35]; *p* < 0.001) for obesity grade I, HR 1.68 (95% CI [1.52, 1.86]; *p* < 0.001) for obesity grade II, and HR 1.92 (95% CI [1.59, 2.33]; *p* < 0.001) for obesity grade III, respectively. The associations were similar for several of the most common underlying causes of death in young age, i.e., respiratory diseases, cardiovascular diseases, and congenital anomalies. The associations were attenuated but remained in the cousin comparison analysis. The main limitation was the potential for residual confounding; the observed associations should not be interpreted as evidence of a causal effect of maternal BMI in early pregnancy on offspring mortality.

**Conclusions:**

Offspring born to mothers with overweight or obesity during early pregnancy had an increased risk of premature mortality up to early adulthood. Given the rising obesity rate in women of childbearing age, our findings underscore the need to better understand the underlying mechanisms for this association. If confirmed by further studies, these results highlight the importance of achieving a healthy maternal BMI prior to conception as a potential strategy to improve child health.

## Author summary

**Why was this study done?:**

- The prevalence of obesity in women of childbearing age has increased worldwide.
- Maternal obesity during pregnancy may have long-term implications for offspring health, but its association with offspring mortality is not well understood.

**What did the researchers do and find?:**

- Using a nationwide cohort of more than 2.6 million live births, we examined whether maternal body mass index (BMI) in early pregnancy was associated with offspring mortality.
- Compared with the reference group with maternal BMI in the normal range, maternal overweight or obesity in early pregnancy was associated with a higher risk of offspring death; the association was stronger with increasing maternal BMI.
- Similar patterns were observed for several common causes of death.
- The associations were attenuated but remained in the cousin comparison analysis.

**What do these findings mean?:**

- These findings suggest that maternal overweight or obesity in early pregnancy may be associated with an increased risk of premature death in offspring up to early adulthood.
- If confirmed by future studies, these findings may have implications for public health efforts to support a healthy BMI before pregnancy.
- A main limitation of the study is that causality cannot be determined, thus the observed associations should not be interpreted as evidence of a causal effect of maternal BMI in early pregnancy on offspring mortality.

## Abstract 3

In this nationwide Swedish cohort study, Hui Wang and colleagues found that higher maternal BMI in early pregnancy was associated with a greater risk of offspring mortality from birth through early adulthood, including deaths related to respiratory and cardiovascular diseases and congenital anomalies.

## Introduction

Obesity has become increasingly prevalent among women of childbearing age worldwide [1]. A recent meta-analysis reported that the global prevalence of obesity in women of childbearing age has reached 16.3%, i.e., it affects approximately one in six pregnancies [2]. Compelling evidence suggests that maternal obesity during pregnancy is associated with several pregnancy complications, including miscarriage, hypertensive disorders of pregnancy, gestational diabetes mellitus, preterm birth, and delivery of a large for gestational age infant [3,4]. Women with these pregnancy complications have increased risks of developing type 2 diabetes mellitus, cardiovascular diseases, and neurological disorders later in life [5,6]. Maternal obesity during pregnancy may be an important determinant also for the long-term health of the offspring, both during childhood and in adulthood [7]. Maternal obesity has been found to be associated with increased risks of obesity [8,9], metabolic disorders [10], cardiovascular diseases [11], and infectious diseases in offspring [12].

Two studies reported that maternal overweight and obesity are associated with an increased risk of infant mortality [13,14]. Knowledge regarding the association between maternal overweight and obesity and the long-term risk of mortality in offspring is, however, limited. To our knowledge, only one study investigated this question and reported that maternal obesity was associated with a 30% increased risk of all-cause mortality in adult offspring [15]. However, due to its relatively small sample size (*n* = 37,709), this study could not analyze dose–response relationships, nor the risk associated with the offspring’s cause-specific death. Further, the role of adverse pregnancy outcomes in this association has not been explored. Pregnancy complications, such as preterm birth and hypertensive disorders of pregnancy, are also associated with increased risks of premature mortality in offspring [16,17]. Finally, it remains unclear whether the observed associations may be confounded by shared familial factors that influence both obesity and mortality.

In this nationwide cohort study, we aimed to analyze the associations of maternal BMI with the risk of all-cause and cause-specific mortality in offspring during childhood, adolescence, and early adulthood, and when taking adverse pregnancy outcomes into consideration. We further assessed the role of confounding by shared familial (genetic or environmental) factors using cousin comparison analysis. We hypothesized that increased maternal BMI in early pregnancy is associated with a higher risk of offspring mortality.

## Methods

### Ethics statement

The study was approved by the Research Ethics Committee at Karolinska Institute in Stockholm (No. 2016/288-31/1, 2021-03315, 2025-04808-2). The requirement for informed consent was waived because the study was based on pseudonymized individual-level data from Swedish national registers and did not involve direct contact with study participants. This study is reported as per the Reporting of Studies Conducted using Observational Routinely-Collected Data (RECORD) guideline (S1 STROBE Checklist).

### Study population and design

The Swedish Medical Birth Register (MBR) includes information on more than 98% of births in Sweden from 1973 onward [18]. The MBR contains data on maternal weight and height at the first prenatal visit since 1982, except for the years 1990 and 1991 [18]. Thus, we restricted our study period to January 1, 1982, through December 31, 2014, without data from 1990 and 1991, and identified all 3,141,610 live births recorded in the MBR during this period [18]. After excluding births with missing (*n* = 531,900) or invalid maternal body mass index (BMI) in early pregnancy (i.e., > 5 standard deviations above or below the mean) (*n* = 3,206), 2,606,684 births remained in the study; a flowchart for participation in the study is shown in S1 Fig. The included and excluded participants were generally comparable with respect to several baseline characteristics; nevertheless, the excluded group was more likely to be born in earlier calendar periods than the group included in the study (S1 Table). Follow-up started on the date of birth and ended on the date of death, emigration (available until December 31, 2014), or December 31, 2023. Using the person-unique national registration numbers of mothers and children, we linked the MBR with the Swedish National Patient Register, the Total Population Register, the Education Register, the Multigeneration Register, and the Cause of Death Register [19–22]. Due to regulations of use of sensitive data in Sweden, the research data analyzed in this study are not openly available. Interested researchers with ethical permit may apply for similar data to the National Board of Health and Welfare and to Statistics Sweden.

### Measures

#### Maternal body mass index.

Information on maternal height and weight during early pregnancy was retrieved from the Medical Birth Register. Height was self-reported, and weight was measured in light clothing at the first prenatal visit, which occurs within the first 14 weeks of gestation for 90% of pregnant women in Sweden [18]. Early pregnancy maternal BMI was calculated as weight in kilograms divided by height in meters squared (kg/m^2^), and categorized according to the World Health Organization’s classification as: underweight (<18.5 kg/m^2^), normal weight (18.5–24.9 kg/m^2^), overweight (25.0–29.9 kg/m^2^), obesity grade I (30.0–34.9 kg/m^2^), obesity grade II (35.0–39.9 kg/m^2^), and obesity grade III (≥40.0 kg/m^2^).

#### All-cause and cause-specific mortality.

The main outcome was all-cause mortality in offspring. Information on the date and underlying cause of death was obtained from the Cause of Death Register [21]. We further categorized deaths based on the underlying cause of death into the most common specific causes of death in this population, i.e., due to congenital anomalies, respiratory diseases, neurological diseases, cardiovascular diseases, cancer, and unnatural causes including suicide and accidents [16]. The International Classification of Diseases codes used to categorize causes of deaths are shown in S2 Table. In Sweden, the reporting of cause of death is mandatory and the accuracy of the underlying causes has been shown to be high [21].

#### Covariates.

Covariates were selected a priori based on previous literature and a directed acyclic graph (S2 Fig). Accordingly, the following covariates, suggested in earlier studies to be associated with both maternal obesity and premature death [11,13,16], were included in the main analyses: maternal characteristics (age at the childbirth in completed years [≤24, 25–29, 30–34, ≥35 years] and smoking status during early pregnancy [yes, no] from MBR, highest attained level of education [≤9 years, primary and lower secondary; 10–12 years, upper secondary; and >12 years, bachelor or higher] from the Education Register, marital status [married/registered partnership or others] and country of birth [Sweden or other country] from the Total Population Register [20], income [categorized based on the tertile distribution of each 10-year interval] from the Register of Incomes and Taxes, history of psychiatric disorders [yes, no] and cardiovascular diseases [yes, no] before the index birth from the Patient Register), birth order [1, 2, ≥3], calendar year of the offspring birth [categorized in four-year intervals], and sex of the offspring. Information on birth order, calendar birth year and sex of the offspring was also from MBR. If information on maternal education and income for the corresponding year was lacking, we used information from the closest year with available data in the five years before study entry. For sensitivity analyses, we also retrieved data on pre-existing diabetes from the MBR (since 1987) and autoimmune disease from the Patient Register [23]. Family history of psychiatric disorders and cardiovascular diseases was defined as the presence of any psychiatric disorders or cardiovascular disease, identified from the Patient Register, in the women’s mother or father, identified from the Multigeneration Register. The International Classification of Diseases (ICD) codes for the discharge diagnoses we searched for are shown in S2 Table).

Perinatal factors included preterm birth, hypertensive disorders of pregnancy, gestational diabetes mellitus, hypoxic-ischemic encephalopathy, and large (above the >97th percentile) or small (under the <3rd percentile) for gestational age. Information on hypertensive disorders of pregnancy and gestational diabetes mellitus were retrieved from the MBR. Data on hypoxic-ischemic encephalopathy was extracted from Patient Register. The ICD codes used for the diseases retrieved in this study are presented in S2 Table. Gestational age at birth was identified from the MBR [18]. Preterm birth was defined as delivery before 37 completed weeks of gestation. Large or small for gestational age was based on the Swedish sex-specific reference curve [24].

### Statistical analysis

Using Cox proportional hazard regression, we estimated hazard ratios (HRs) and 95% confidence intervals (95% CI) for all-cause and cause-specific mortality in offspring of underweight mothers (BMI < 18.5), overweight mothers (BMI of 25.0–29.9), and mothers in obesity grade I (BMI of 30.0–34.9), II (BMI of 35.0–39.9), and III (BMI ≥ 40.0) compared with the offspring of normal weight mothers (BMI of 18.5–24.9), with adjustment for the covariates described above. Time since birth was used as the underlying time scale. The proportional hazard assumption was assessed using Schoenfeld residuals and by visual inspection of log-log survival plots. In all Cox models, we specified the robust sandwich estimate of the covariance matrix to account for sequential births by the same mother. We tested linear trends for the association between maternal BMI and mortality in offspring by introducing a variable representing ordinal categories of BMI as a continuous predictor. Given the different mortality rates across age [25], we split the follow-up time by attained age to examine whether the associations vary by age group. We created age groups based on developmental periods, i.e., ≤ 1 years (infancy), > 1 to ≤5 years (childhood), > 5 to ≤18 years (adolescence), and >18 years (adulthood). To assess the nonlinear relationship between maternal BMI and offspring mortality, we used a restricted cubic spline with 4 knots placed at the 5th, 35th, 65th, and 95th percentiles.

In the analyses with cause-specific mortality, we considered deaths from other causes as competing events and censored participants on the date of death from other causes. In addition, to ensure adequate statistical power when examining cause-specific mortality, we combined obesity (grade I, II, and III) into one group.

To account for the confounding by unmeasured shared familial factors, we conducted cousin comparison analyses. Since the exposure was maternal BMI during pregnancy, we employed maternal parallel cousin comparison analyses, which compares individuals who share biological grandparents [26]. Because cousins are born to different mothers, cousin comparisons are less affected by processes specific to pregnancy order, carryover effects across pregnancies, and event-dependent reproductive behaviors that may affect sibling analyses. In addition, cousin analyses retain a substantially larger and less selected sample than would be possible in case of sibling-discordant analyses for a relatively rare outcome such as offspring mortality. This approach allows for control of unmeasured confounding factors, such as genetic and environmental influences, that are stable within extended families. Stratified Cox regression models were performed, with each cousin pair treated as a separate stratum, and adjusted for the same covariates as those used in primary analysis. In cousin comparison analyses, only cousins sets discordant for the exposure are informative for estimating the association; within those sets, outcome variation determines whether they contribute to the partial likelihood in the Cox model.

To explore the potential mediating role of adverse pregnancy outcomes in the association between maternal obesity and offspring mortality, we performed mediation analyses [27]. Mediation analyses were performed using the cmest function in the CMAverse package based on the counterfactual framework, allowing the decomposition of the total effect into the natural direct effect and the natural indirect effect. The survival outcome was modeled using Cox proportional hazards regression, with time-to-event and event indicator specified explicitly, while the binary mediator was modeled using logistic regression. Interaction terms between exposure and mediator were also included, and 95% CI were obtained using bootstrap resampling. The models were adjusted for covariates consistent with those in the primary analysis. Potential mediators were selected based on their biological and clinical relevance to maternal obesity, which was the exposure pattern most clearly associated with increased offspring mortality in our data. The mediators we examined were preterm birth, hypertensive disorder of pregnancy, gestational diabetes mellitus, large for gestational age, and small for gestational age. We first assessed each mediator individually and then performed an additional combined mediation analysis including all measured perinatal factors as multiple mediators in the same model to estimate their joint mediating effect.

To test the robustness of our findings, we conducted several sensitivity analyses. First, as approximately 17% of offspring were excluded from the primary analysis because of missing data on maternal BMI, we imputed missing values for maternal BMI and all the covariates used in the main analyses through multiple imputation by chained equations [28]. We created 20 imputed datasets and performed Cox regression analyses separately for each dataset. The results were then combined using Rubin’s rules to produce the final summary estimates [28]. Furthermore, we restricted the cohort to offspring born from 1992 onward—a period with more complete ascertainment of maternal BMI—to evaluate whether the main findings were influenced by historical patterns of missing BMI data. Second, to assess the potential impact of incomplete emigration data after 2014, we additionally conducted a sensitivity analysis restricting follow-up to 2014. Third, to examine whether the association between maternal BMI in early pregnancy and offspring mortality during infancy was driven mainly by deaths in the neonatal period, we further divided infancy (0–1 year) into the neonatal period (0–28 days) and the post-neonatal period of infancy (29–365 days). Fourth, to assess whether pre-existing maternal diabetes, autoimmune disease, family history of psychiatric disorders and cardiovascular diseases, may confound the studied association we performed additional analyses by adding these factors to the main model both sequentially and simultaneously (during a period with full data availability). In analyses adjusting for autoimmune disease, the study population was restricted to offspring born during 2001–2014 because ascertainment of autoimmune disease was considered more reliable during this period [23]. Fifth, to further explore the role of perinatal factors in the association between maternal BMI in early pregnancy and offspring mortality, we sequentially excluded offspring from pregnancies affected by each of the perinatal factors, namely, preterm birth, hypertensive disorders of pregnancy, gestational diabetes mellitus, hypoxic-ischemic encephalopathy, and large or small for gestational age from the analyses, one at a time. We also performed a separate analysis in which offspring from pregnancies affected by any of these perinatal factors were excluded. Lastly, we also performed stratified analyses by maternal country of birth, maternal education, calendar period of birth year, and parity to investigate potential effect modification by these variables. This study was guided by a prespecified analysis plan, which is provided as Analysias pln in S1 File. No artificial intelligence tools or technologies were used in the preparation of the manuscript.

### Additional analyses conducted during revision

Compared with the original analysis plan (Analysis plan in S1 File), additional analyses were conducted during revision to evaluate the robustness of the findings and to address issues raised during peer review. These analyses included restricting the cohort to offspring born from 1992 onward; restricting follow-up to 31 December 2014; subdividing deaths before 1 year of age into neonatal and post-neonatal deaths; further adjusting for pre-existing maternal diabetes and autoimmune disease; excluding offspring from pregnancies affected by any measured perinatal factor; stratifying analyses by maternal country of birth; and using a composite perinatal mediator, defined as the presence of any measured perinatal factor.

## Results

During the median follow-up of 22.4 years (interquartile range 14.9–30.7 years), 21,981 offsprings died, 8,135 (37.0%) during infancy, 1854 (8.4%) during childhood, 3,103 (14.1%) during adolescence, and 8,889 (40.5%) during adulthood. Offspring mortality increased with male sex, higher birth order, earlier birth, younger maternal age at childbirth, lower maternal education, maternal psychiatric disorders and cardiovascular diseases before childbirth, maternal country of birth outside Sweden, maternal smoking during pregnancy, preterm birth and hypertensive disorders of pregnancy (S3 Table). During the study period, the prevalence of maternal overweight (BMI of 25.0–29.9) in early pregnancy increased from 10.7% in 1982 to 25.2% in 2014, while the corresponding increase for maternal obesity (BMI ≥ 30.0) was from 2.0% to 12.9% (S4 Table). Maternal mean BMI in early pregnancy was 23.8 kg/m^2^ (standard deviation 4.2). Mothers who were overweight or obese were more likely to have higher parity, older age, lower education, and a history of psychiatric disorders, cardiovascular diseases (Table 1).

**Table 1:** Baseline characteristics of the study population according to maternal body mass index during early pregnancy.

Characteristics	Maternal body mass index, kg/m^2^ N (%)					
	<18.5	18.5–24.9	25.0–29.9	30.0–34.9	35.0–39.9	≥40
	Underweight	Normal weight	Overweight	Obesity grade I	Obesity grade II	Obesity grade III
	*N* = 99,302	*N* = 1,719,560	*N* = 563,076	*N* = 166,462	*N* = 46,009	*N* = 12,275
Sex of the offspring						
Male	50,490 (50.8)	883,603 (51.4)	289,699 (51.4)	85,682 (51.5)	23,704 (51.5)	6,187 (50.4)
Female	48,812 (49.2)	835,957 (48.6)	273,377 (48.6)	80,780 (48.5)	22,305 (48.5)	6,088 (49.6)
Birth order of the offspring						
1	48,574 (48.9)	772,202 (44.9)	213,816 (38.0)	57,633 (34.6)	15,725 (34.2)	4,168 (34.0)
2	35,301 (35.5)	624,859 (36.3)	208,365 (37.0)	60,433 (36.3)	16,417 (35.7)	4,360 (35.5)
≥3	15,427 (15.5)	322,499 (18.8)	140,895 (25.0)	48,396 (29.1)	13,867 (30.1)	3,747 (30.5)
Calendar year of offspring birth						
1982–1985	20,899 (21.0)	200,010 (11.6)	29,589 (5.3)	5,164 (3.1)	388 (0.8)	31 (0.3)
1986–1989	24,241 (24.4)	229,075 (13.3)	38,634 (6.9)	6,756 (4.1)	457 (1.0)	16 (0.1)
1992–1995	12,164 (12.2)	251,037 (14.6)	75,083 (13.3)	18,729 (11.3)	4,499 (9.8)	939 (7.6)
1996–1999	8,138 (8.2)	192,612 (11.2)	70,536 (12.5)	19,679 (11.8)	5,100 (11.1)	1,253 (10.2)
2000–2003	7,604 (7.7)	196,042 (11.4)	78,520 (13.9)	23,862 (14.3)	6,964 (15.1)	1,833 (14.9)
2004–2007	8,165 (8.2)	218,498 (12.7)	88,673 (15.7)	28,447 (17.1)	8,909 (19.4)	2,555 (20.8)
2008–2011	9,959 (10.0)	246,314 (14.3)	102,126 (18.1)	34,991 (21.0)	10,798 (23.5)	3,150 (25.7)
2012–2014	8,132 (8.2)	185,972 (10.8)	79,915 (14.2)	28,834 (17.3)	8,894 (19.3)	2,498 (20.4)
Maternal age (year)						
≤24	32,442 (32.7)	323,059 (18.8)	93,293 (16.6)	28,041 (16.8)	7,474 (16.2)	1,929 (15.7)
25–29	35,110 (35.4)	587,447 (34.2)	178,472 (31.7)	52,375 (31.5)	14,561 (31.6)	3,812 (31.1)
30–34	23,048 (23.2)	541,708 (31.5)	178,873 (31.8)	51,101 (30.7)	14,166 (30.8)	3,858 (31.4)
≥35	8,702 (8.8)	267,346 (15.5)	112,438 (20.0)	34,945 (21.0)	9,808 (21.3)	2,676 (21.8)
Maternal education (year)						
≤9	20,728 (20.9)	214,682 (12.5)	82,902 (14.7)	29,584 (17.8)	8,435 (18.3)	2,438 (19.9)
10–12	46,005 (46.3)	770,234 (44.8)	273,845 (48.6)	86,871 (52.2)	25,354 (55.1)	6,885 (56.1)
>12	29,037 (29.2)	699,330 (40.7)	192,745 (34.2)	45,597 (27.4)	11,048 (24.0)	2,685 (21.9)
Missing	3,532 (3.6)	35,314 (2.1)	13,584 (2.4)	4,410 (2.6)	1,172 (2.5)	267 (2.2)
Maternal income						
Lowest tertile	45,178 (45.5)	586,221 (34.1)	187,366 (33.3)	57,118 (34.3)	15,837 (34.4)	4,422 (36.0)
Middle tertile	30,485 (30.7)	570,610 (33.2)	192,908 (34.3)	58,631 (35.2)	16,700 (36.3)	4,407 (35.9)
Highest tertile	23,427 (23.6)	560,059 (32.6)	181,892 (32.3)	50,479 (30.3)	13,434 (29.2)	3,433 (28.0)
Missing	212 (0.2)	2,670 (0.2)	910 (0.2)	234 (0.1)	38 (0.1)	13 (0.1)
Maternal country of birth						
Sweden	78,215 (78.8)	1,439,093 (83.7)	449,227 (79.8)	130,963 (78.7)	37,210 (80.9)	10,125 (82.5)
Other country	21,045 (21.2)	279,405 (16.2)	113,432 (20.1)	35,353 (21.2)	8,754 (19.0)	2,128 (17.3)
Missing	42 (0)	1,062 (0.1)	417 (0.1)	146 (0.1)	45 (0.1)	22 (0.2)
Maternal marital status						
Married or register partnership	46,756 (47.0)	758,754 (44.2)	233,726 (41.5)	65,027 (39.1)	16,915 (36.8)	4,420 (36.0)
Others	50,710 (51.0)	910,555 (53.0)	304,261 (54.0)	92,144 (55.4)	26,063 (56.6)	6,981 (56.9)
Missing	1,836 (1.8)	50,251 (2.9)	25,089 (4.4)	9,291 (5.6)	3,031 (6.6)	874 (7.1)
Maternal smoking status during early pregnancy						
No	71,418 (71.9)	1,432,808 (83.3)	477,165 (84.7)	139,339 (83.7)	38,433 (83.5)	10,235 (83.4)
Yes	23,161 (23.3)	234,315 (13.6)	73,451 (13.0)	24,033 (14.4)	6,869 (14.9)	1,856 (15.1)
Missing	4,723 (4.8)	52,437 (3.0)	12,460 (2.2)	3,090 (1.9)	707 (1.5)	184 (1.5)
Maternal psychiatric disorders before childbirth						
No	92,768 (93.4)	1,620,130 (94.2)	521,987 (92.7)	150,954 (90.7)	40,881 (88.9)	10,689 (87.1)
Yes	6,534 (6.6)	99,430 (5.8)	41,089 (7.3)	15,508 (9.3)	5,128 (11.1)	1,586 (12.9)
Maternal cardiovascular diseases before childbirth						
No	97,563 (98.2)	1,680,824 (97.7)	546,381 (97.0)	160,481 (96.4)	44,087 (95.8)	11,722 (95.5)
Yes	1,739 (1.8)	38,736 (2.3)	16,695 (3.0)	5,981 (3.6)	1,922 (4.2)	553 (4.5)
Maternal pre-existing diabetes						
No	71,924 (72.4)	1,456,670 (84.7)	521,066 (92.5)	158,281 (95.1)	44,972 (97.7)	12,039 (98.1)
Yes	70 (0.1)	5,047 (0.3)	3,357 (0.6)	1,413 (0.8)	526 (1.1)	200 (1.6)
Missing	27,308 (27.5)	257,843 (15.0)	38,653 (6.9)	6,768 (4.1)	511 (1.1)	36 (0.3)
Maternal autoimmune disease before childbirth						
No	29,693 (29.9)	744,344 (43.3)	304,824 (54.1)	100,481 (60.4)	30,570 (66.4)	8,565 (69.8)
Yes	2,298 (2.3)	56,528 (3.3)	26,322 (4.7)	10,266 (6.2)	3,523 (7.7)	1,122 (9.1)
Missing	67,311 (67.8)	918,688 (53.4)	231,930 (41.2)	55,715 (33.5)	11,916 (25.9)	2,588 (21.1)
Maternal family history of cardiovascular diseases						
No	65,209 (65.7)	1,082,203 (62.9)	317,883 (56.4)	88,557 (53.2)	24,155 (52.5)	6,227 (50.7)
Yes	18,694 (18.8)	436,149 (25.4)	160,780 (28.6)	51,804 (31.1)	15,616 (33.9)	4,561 (37.2)
Missing	15,399 (15.5)	201,208 (11.7)	84,413 (15.0)	26,101 (15.7)	6,238 (13.6)	1,487 (12.1)
Maternal family history of psychiatric disorders						
No	72,840 (73.4)	1,329,293 (77.3)	412,348 (73.2)	118,541 (71.2)	33,167 (72.1)	8,865 (72.2)
Yes	11,063 (11.1)	189,059 (11.0)	66,315 (11.8)	21,820 (13.1)	6,604 (14.4)	1,923 (15.7)
Missing	15,399 (15.5)	201,208 (11.7)	84,413 (15.0)	26,101 (15.7)	6,238 (13.6)	1,487 (12.1)
Preterm birth						
No	92,320 (93.0)	1,626,865 (94.6)	530,893 (94.3)	155,593 (93.5)	42,566 (92.5)	11,283 (91.9)
Yes	6,817 (6.9)	91,383 (5.3)	31,923 (5.7)	10,790 (6.5)	3,427 (7.4)	986 (8.0)
Missing	165 (0.1)	1,312 (<0.1)	260 (<0.1)	79 (<0.1)	16 (<0.1)	6 (<0.1)
Hypertensive disorders of pregnancy						
No	97,306 (98.0)	1,669,811 (97.1)	536,500 (95.3)	154,889 (93.0)	41,846 (91.0)	11,005 (89.7)
Yes	1996 (2.0)	49,749 (2.9)	26,576 (4.7)	11,573 (7.0)	4,163 (9.0)	1,270 (10.3)
Gestational diabetes mellitus						
No	71,694 (72.2)	1,454,746 (84.6)	518,274 (92.0)	155,582 (93.5)	43,461 (94.6)	11,518 (93.8)
Yes	300 (0.3)	6,971 (0.4)	6,149 (1.1)	4,112 (2.5)	2,037 (4.4)	721 (5.9)
Missing	27,308 (27.5)	257,843 (15.0)	38,653 (6.9)	6,768 (4.1)	511 (1.1)	36 (0.3)
Large for gestational age (>97th percentile)						
No	97,577 (98.3)	1,660,753 (96.6)	527,452 (93.7)	151,820 (91.2)	40,746 (88.6)	10,594 (86.3)
Yes	1,218 (1.2)	52,417 (3.0)	33,896 (6.0)	14,158 (8.5)	5,126 (11.1)	1,637 (13.3)
Missing	507 (0.5)	6,390 (0.4)	1,728 (0.3)	484 (0.3)	137 (0.3)	44 (0.4)
Small for gestational age (>3^rd^ percentile)						
No	92,534 (93.2)	1,654,119 (96.2)	545,001 (96.8)	160,969 (96.7)	44,435 (96.6)	11,821 (96.3)
Yes	6,261 (6.3)	59,051 (3.4)	16,347 (2.9)	5,009 (3.0)	1,437 (3.1)	410 (3.3)
Missing	507 (0.5)	6,390 (0.4)	1,728 (0.3)	484 (0.3)	137 (0.3)	44 (0.4)
Hypoxic-ischemic encephalopathy						
No	39,634 (39.9)	986,376 (57.4)	401,390 (71.3)	130,932 (78.7)	39,453 (85.8)	11,001(89.6)
Yes	5 (<0.1)	387 (<0.1)	249 (<0.1)	98 (<0.1)	41(<0.1)	17(0.1)
Missing	59,663 (60.1)	732,797(42.6)	161,437 (18.7)	35,432 (21.3)	6,515 (14.2)	1,257 (10.2)

Data on pre-existing diabetes, gestational diabetes mellitus, hypoxic-ischemic encephalopathy, and autoimmune disease are shown as missing for births before 1987, 1987, 1997, and 2001, respectively.

**Fig 1:**
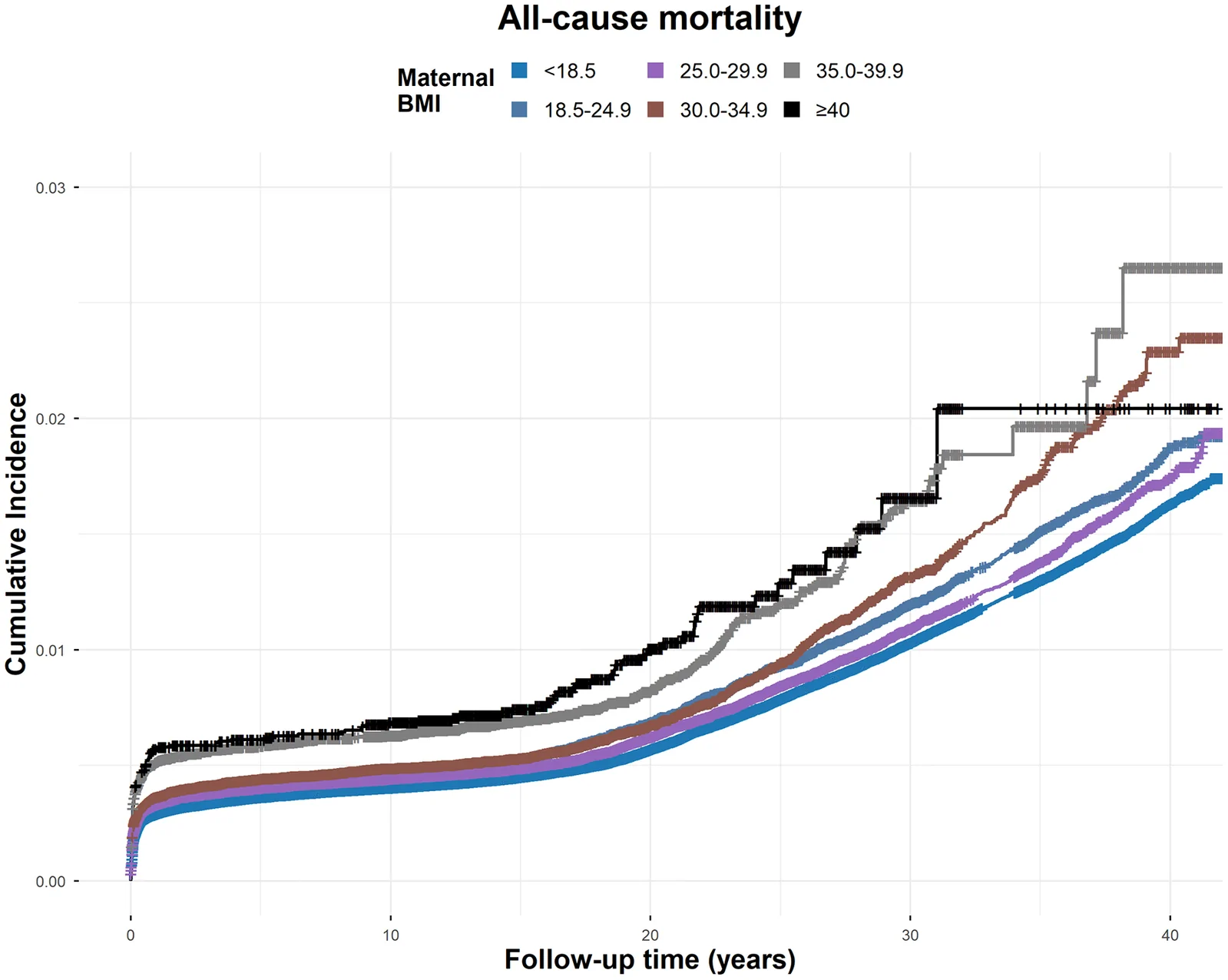
Cumulative incidence of all-cause mortality in offspring by maternal body mass index in early pregnancy.

The cumulative incidence of offspring mortality according to maternal BMI category in early pregnancy is shown in Fig 1. The proportional hazards assumption was supported by the Schoenfeld residuals test (*p* value = 0.08) and by visual inspection of log–log survival plots (S3 Fig). Maternal BMI in early pregnancy was positively associated with all-cause mortality in offspring: HR 1.03 (95% CI [1.02,1.03]; *p* < 0.001) per 1 unit increase in BMI. Offspring of mothers with overweight, obesity grade I, obesity grade II, and obesity grade III had increasingly higher risks of mortality than offspring of mothers with normal BMI; the corresponding HRs were HR 1.11 (95% CI [1.07, 1.15]; *p* < 0.001), HR 1.27 (95% CI [1.20, 1.35]; *p* < 0.001), HR 1.68 (95% CI [1.52, 1.86]; *p* < 0.001), and HR 1.92 (95% CI [1.59, 2.33]; *p* < 0.001), respectively. Restricted cubic spline analyses suggested a J-shaped association between maternal BMI in early pregnancy and offspring mortality (S4 Fig). The curve was relatively flat at lower BMI levels, with risk increasing from approximately 23 kg/m^2^ onward in a dose-response manner. We observed that the associations between maternal BMI and mortality during infancy, adolescence, and adulthood were consistent with those identified over the entire follow-up period, whereas no significant association was observed during childhood (Table 2). The results were similar when restricting the cohort to offspring born in 1992 or later (Table 3) or when additional adjustment for maternal pre-existing diabetes or autoimmune disease (among offspring born in 2001 or later), and for family history of psychiatric disorders or cardiovascular disease (among offspring with such data) (Table 4).

**Table 2:** Associations between maternal body mass index in early pregnancy and risks of all-cause mortality in offspring, overall and stratified by attained age of the offspring, adjusted hazard ratios and 95% confidence intervals born in or after 1982.

	Number	Case (Rate*)	Adjusted HR [95% CI]	*p* value
Per BMI unit increase			1.03 [1.02, 1.03]	<0.001
BMI category				
<18.5	99,302	1,165 (4.2)	1.02 [0.96, 1.09]	0.572
18.5–24.9	1,719,560	14,557 (3.5)	1.00 [ref]	NA
25.0–29.9	563,076	4,368 (3.6)	1.11 [1.07, 1.15]	<0.001
30.0–34.9	166,462	1,355 (4.1)	1.27 [1.20, 1.35]	<0.001
35.0–39.9	46,009	420 (5.0)	1.68 [1.52, 1.86]	<0.001
≥40	12,275	116 (5.4)	1.92 [1.59, 2.33]	<0.001
Attained age ≤1 years				
<18.5	99,302	355 (1.3)	1.07 [0.95, 1.20]	0.256
18.5–24.9	1,719,560	5,001 (1.2)	1.00 [ref]	NA
25.0–29.9	563,076	1,865 (1.6)	1.22 [1.15, 1.29]	<0.001
30.0–34.9	166,462	606 (1.8)	1.35 [1.23, 1.47]	<0.001
35.0–39.9	46,009	237 (2.8)	2.06 [1.80, 2.37]	<0.001
≥40	12,275	71 (3.3)	2.44 [1.91, 3.12]	<0.001
Attained age >1 to ≤5 years				
<18.5	98,737	66 (0.2)	0.77 [0.59, 1.00]	0.052
18.5–24.9	1,711,775	1,229 (0.3)	1.00 [ref]	NA
25.0–29.9	560,474	395 (0.3)	0.98 [0.87, 1.11]	0.768
30.0–34.9	165,680	128 (0.4)	1.13 [0.93, 1.38]	0.208
35.0–39.9	45,714	32 (0.4)	1.08 [0.75, 1.57]	0.676
≥40	12,188	NA	NA	NA
Attained age > 5 to ≤18 years				
<18.5	97,839	163 (0.6)	1.18 [1.00, 1.40]	0.053
18.5–24.9	1,698,439	2,005 (0.5)	1.00 [ref]	NA
25.0–29.9	556,382	655 (0.6)	1.10 [1.00, 1.21]	0.049
30.0–34.9	164,510	203 (0.6)	1.19 [1.02, 1.39]	0.031
35.0–39.9	45,411	55 (0.7)	1.25 [0.94, 1.67]	0.130
≥40	12,116	22 (1.0)	2.23 [1.46, 3.41]	<0.001
Attained age >18 years				
<18.5	74,708	581 (2.4)	0.98 [0.89, 1.07]	0.643
18.5–24.9	1,149,173	6,322 (1.8)	1.00 [ref]	NA
25.0–29.9	328,201	1,453 (1.6)	1.03 [0.97, 1.09]	0.336
30.0–34.9	85,803	418 (1.9)	1.29 [1.16, 1.43]	<0.001
35.0–39.9	21,128	96 (1.9)	1.62 [1.32, 2.00]	<0.001
≥40	5,113	19 (1.6)	1.40 [0.85, 2.28]	0.184

HR, adjusted hazard ratio; CI, confidence interval; BMI, body mass index; NA, indicate less than 5 cases in a cell; ^*^Rate per 10,000 person-years; Models were adjusted for offspring age (time since birth as the underlying time scale), calendar year of birth, sex, birth order, and maternal characteristics (age, education, and income at the childbirth, marital status, country of birth, smoking status in early pregnancy, psychiatric disorders and cardiovascular diseases before the childbirth); *P* values were calculated using 2-sided Wald tests from the Cox proportional hazards models.

**Table 3:** Associations between maternal body mass index in early pregnancy and risks of all-cause mortality in offspring, overall and stratified by attained age of the offspring, adjusted hazard ratios and 95% confidence intervals, among the individuals born in or after 1992.

Maternal BMI, kg/m^2^	Number	Case (Rate*)	Adjusted HR [95% CI]
Per BMI unit increase			1.03 [1.02, 1.03]
BMI category			
<18.5	54,162	350 (3.2)	1.05 [0.94, 1.18]
18.5–24.9	1,290,475	7,247 (2.8)	1.00 [ref]
25.0–29.9	494,853	3,161 (3.3)	1.17 [1.12, 1.22]
30.0–34.9	154,542	1,073 (3.8)	1.29 [1.20, 1.38]
35.0–39.9	45,164	394 (4.9)	1.71 [1.54, 1.91]
≥40	12,228	115 (5.4)	1.96 [1.61, 2.37]
Attained age ≤1 years			
<18.5	54,162	144 (1.3)	1.01 [0.85, 1.21]
18.5–24.9	1,290,475	3,185 (1.2)	1.00 [ref]
25.0–29.9	494,853	1,562 (1.6)	1.27 [1.19, 1.35]
30.0–34.9	154,542	543 (1.9)	1.38 [1.25, 1.52]
35.0–39.9	45,164	229 (2.8)	2.08 [1.80, 2.39]
≥40	12,228	70 (3.3)	2.43 [1.90, 3.12]
Attained age >1 to ≤5 years			
<18.5	53,893	38 (0.3)	1.09 [0.78, 1.54]
18.5–24.9	1,285,152	778 (0.3)	1.00 [ref]
25.0–29.9	492,633	324 (0.3)	1.03 [0.90, 1.18]
30.0–34.9	153,831	109 (0.4)	1.09 [0.88, 1.35]
35.0–39.9	44,878	31 (0.4)	1.10 [0.75, 1.60]
≥40	12,142	4 (0.2)	0.58 [0.22, 1.55]
Attained age >5 to ≤18 years			
<18.5	53,315	63 (0.6)	1.15 [0.89, 1.50]
18.5–24.9	1,274,783	1,222 (0.5)	1.00 [ref]
25.0–29.9	488,933	530 (0.6)	1.13 [1.02, 1.26]
30.0–34.9	152,745	182 (0.6)	1.25 [1.06, 1.47]
35.0–39.9	44,584	51 (0.6)	1.25 [0.93, 1.69]
≥40	12,071	22 (1.0)	2.26 [1.48, 3.46]
Attained age > 18 years			
< 18.5	30,659	105 (1.3)	1.03 [0.83, 1.26]
18.5–24.9	729,190	2,062 (1.1)	1.00 [ref]
25.0–29.9	261,253	745 (1.1)	1.08 [0.99, 1.18]
30.0–34.9	74,131	239 (1.3)	1.26 [1.10, 1.45]
35.0–39.9	20,309	83 (1.7)	1.67 [1.33, 2.09]
≥40	5,069	19 (1.6)	1.45 [0.89, 2.38]

HR, hazard ratio; CI, confidence interval; BMI, body mass index; ^*^Rate per 10,000 person-years; Models were adjusted for offspring age (time since birth as the underlying time scale), calendar year of birth, sex, birth order, and maternal characteristics (age, education, and income at the child’s birth, marital status, country of birth, smoking status in early pregnancy, psychiatric disorders and cardiovascular diseases before the childbirth; P values were calculated using 2-sided Wald tests from the Cox proportional hazards models.

**Table 4:** Association between maternal body mass index in early pregnancy and risk of all-cause mortality in offspring, adjusted hazard ratios and 95% confidence intervals, with inclusion of further potential confounders in addition to those in the main model.

Maternal body mass index, kg/m^2^	Adjusted hazard ratios [95% confidence intervals]				
	Further adjusted for Pre-existing diabetes	Further adjusted for autoimmune disease	Further adjusted for family history of psychiatric disorders	Further adjusted for family history of cardiovascular diseases	Further adjusted for all these potential confounders
	*N* = 2,094,175	*N* = 1,193,512	*N* = 2,127,282	*N* = 2,127,282	*N* = 1,024,468
<18.5	1.04 [0.96, 1.13]	0.91 [0.75, 1.12]	1.03 [0.96, 1.10]	1.03 [0.96, 1.10]	0.92 [0.73, 1.16]
18.5–24.9	1.00 [ref]	1.00 [ref]	1.00 [ref]	1.00 [ref]	1.00 [ref]
25.0–29.9	1.14 [1.09, 1.19]	1.20 [1.12, 1.28]	1.09 [1.05, 1.13]	1.09 [1.05, 1.13]	1.19 [1.10, 1.28]
30.0–34.9	1.29 [1.21, 1.37]	1.31 [1.18, 1.45]	1.27 [1.20, 1.36]	1.27 [1.20, 1.36]	1.30 [1.16, 1.45]
35.0–39.9	1.69 [1.52, 1.88]	1.78 [1.53, 2.06]	1.67 [1.50, 1.87]	1.68 [1.50, 1.87]	1.77 [1.51, 2.08]
40	1.94 [1.60, 2.34]	1.92 [1.48, 2.47]	1.92 [1.57, 2.35]	1.92 [1.57, 2.35]	1.91 [1.45, 2.51]

Main models were adjusted for offspring age (time since birth as the underlying time scale), calendar year of birth, sex, birth order, and maternal characteristics (age, education, and income at the child’s birth, marital status, country of birth, smoking status in early pregnancy, psychiatric disorders and cardiovascular diseases before the childbirth). In analyses with further adjustment for pre-existing diabetes and autoimmune disease, the study population was restricted to offspring born during 1987–2014 and 2001–2014, respectively. In analyses adjusted for all these potential confounders, the study population was restricted to offspring born during 2001–2014.

For the cousin comparison analysis, we identified 980,355 individuals nested within 302,446 extended families. As shown in Table 5, the results from the cousin comparison analysis were directionally consistent with the main analysis; the point estimates were generally lower, and the CIs were wider in the cousin comparison than in the main analysis. In the cousin comparison analysis, offspring of mothers with obesity grade II and obesity grade III had higher risks of mortality than offspring of mothers with the reference category, with HR 1.58 (95% CI [1.23, 2.03]; *p* < 0.001) and HR 1.68 (95% CI [1.06, 2.68]; *p* = 0.028), respectively. The corresponding estimates were HR 1.04 (95% CI [0.96, 1.13); *p* = 0.368) for overweight and HR 1.14 (95% CI [0.98, 1.32]; *p* = 0.086) for obesity grade I.

**Table 5:** Associations between maternal body mass index in early pregnancy and risks of all-cause mortality in offspring, overall and stratified by attained age of the offspring, adjusted hazard ratios and 95% confidence intervals, cousin comparison analysis.

	Number	Case (Rate*)	Adjusted HR [95% CI]	*p* value
Per BMI unit increase			1.02 [1.01, 1.03]	<0.001
BMI category				
<18.5	37,813	478 (4.2)	0.95 [0.82, 1.11]	0.537
18.5–24.9	665,674	6,116 (3.6)	1.00 [ref]	NA
25.0–29.9	200,503	1,659 (3.6)	1.04 [0.96, 1.13]	0.368
30.0–34.9	56,695	500 (4.2)	1.14 [0.98, 1.32]	0.086
35.0–39.9	15,523	151 (5.0)	1.58 [1.23, 2.03]	<0.001
≥40	4,147	42 (5.5)	1.68 [1.06, 2.68]	0.028
Attained age ≤1 years				
<18.5	37,813	145 (1.3)	1.05 [0.82, 1.35]	0.705
18.5–24.9	665,674	2,036 (1.2)	1.00 [ref]	NA
25.0–29.9	200,503	709 (1.6)	1.09 [0.96, 1.25]	0.178
30.0–34.9	56,695	215 (1.8)	1.10 [0.89, 1.36]	0.375
35.0–39.9	15,523	83 (2.7)	1.89 [1.34, 2.67]	<0.001
≥40	4,147	26 (3.4)	1.97 [1.09, 3.59]	0.025
Attained age >1 to ≤5 years				
<18.5	37,650	19 (0.2)	0.56 [0.29, 1.07]	0.080
18.5–24.9	663,378	478 (0.3)	1.00 [ref]	NA
25.0–29.9	199,722	133 (0.3)	0.85 [0.64, 1.12]	0.245
30.0–34.9	56,462	39 (0.3)	0.70 [0.42, 1.16]	0.163
35.0–39.9	15,435	7 (0.2)	0.41 [0.16, 1.06]	0.067
≥40	4,119	NA	NA	NA
Attained age >5 to ≤18 years				
<18.5	37,544	60 (0.5)	1.19 [0.81, 1.75]	0.388
18.5–24.9	661,377	842 (0.5)	1.00 [ref]	NA
25.0–29.9	199,212	233 (0.5)	1.05 [0.85, 1.31]	0.635
30.0–34.9	56,327	64 (0.5)	1.05 [0.71, 1.55]	0.791
35.0–39.9	15,408	25 (0.8)	1.65 [0.89, 3.07]	0.114
≥40	4,112	6 (0.8)	2.71 [0.76, 9.72]	0.125
Attained age > 18 years				
<18.5	31,514	254 (2.4)	0.89 [0.70, 1.14]	0.359
18.5–24.9	490,616	2,760 (1.9)	1.00 [ref]	NA
25.0–29.9	131,851	584 (1.6)	0.99 [0.84, 1.16]	0.903
30.0–34.9	33,676	182 (2.0)	1.54 [1.16, 2.03]	0.002
35.0–39.9	8,128	36 (1.8)	1.74 [1.00, 3.04]	0.053
≥40	1,977	8 (1.7)	1.42 [0.46, 4.42]	0.548

HR, adjusted hazard ratio; CI, confidence interval; BMI, body mass index; NA, indicate less than 5 cases in a cell; ^*^Rate per 10,000 person-years; Models were adjusted for offspring age (time since birth as the underlying time scale), calendar year of birth, sex, birth order, and maternal characteristics (age, education, and income at the childbirth, marital status, country of birth, smoking status in early pregnancy, psychiatric disorders and cardiovascular diseases before the childbirth). P values were calculated using 2-sided Wald tests from the Cox proportional hazards models.

**Fig 2:**
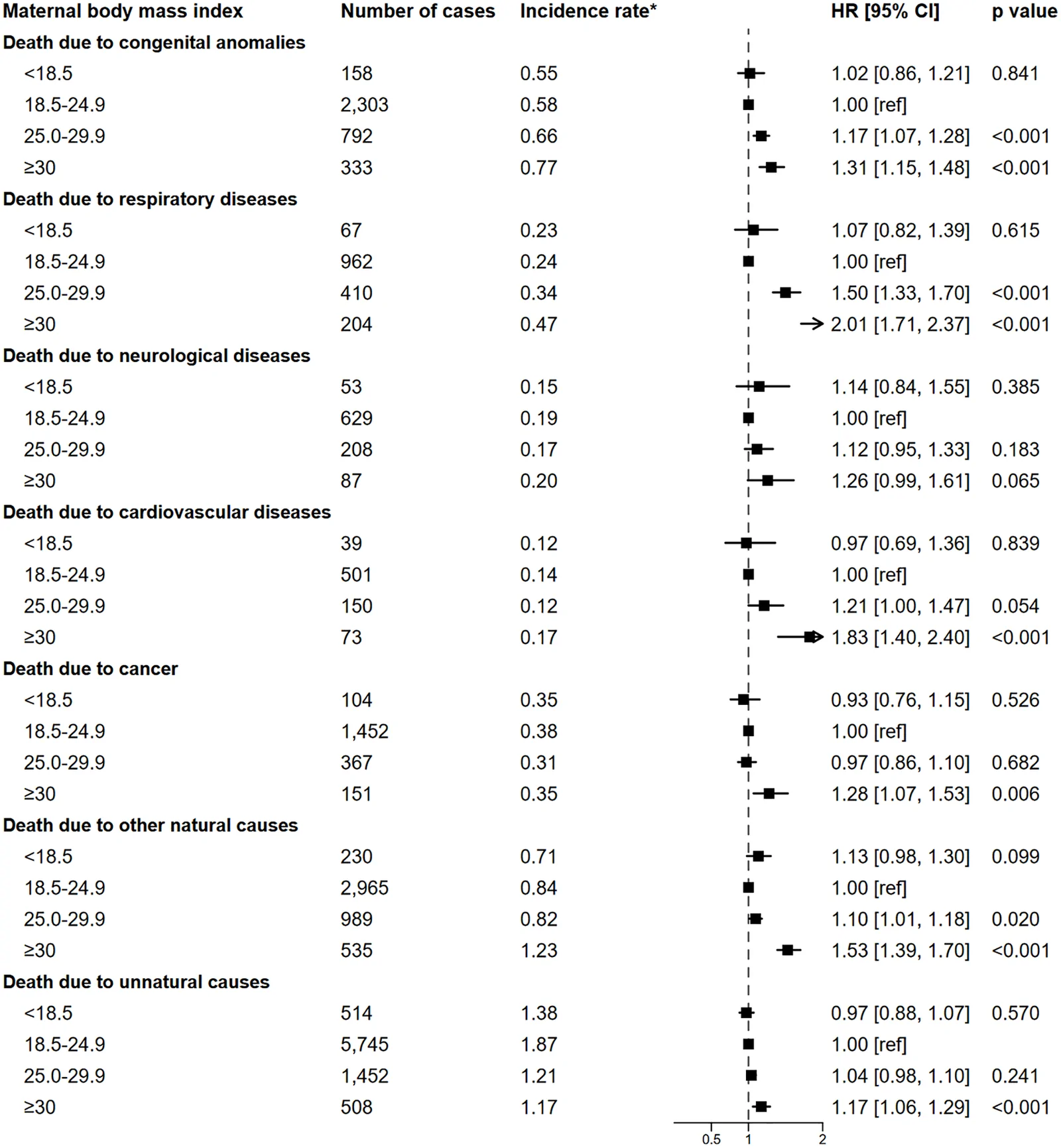
Associations between maternal body mass index during early pregnancy and risk of cause-specific mortality in offspring, adjusted hazard ratio and 95% confidence intervals. ^*^Rate per 10,000 person-years; HR, hazard ratios; CI, confidence intervals. Models were adjusted for offspring age, calendar year of birth, sex, birth order, and maternal characteristics (age, education, and income at the childbirth, marital status, country of birth, smoking status in early pregnancy, psychiatric disorders and cardiovascular diseases before the childbirth).

Associations between maternal overweight and obesity and cause-specific mortality in offspring are shown in Fig 2. Compared with offspring of normal weight mothers, those with obese mothers in early pregnancy had higher risks of offspring mortality due to congenital anomalies (HR 1.31, 95% CI [1.15, 1.48]; *p* < 0.001), respiratory diseases (HR 2.01, 95% CI [1.71, 2.37]; *p* < 0.001), cardiovascular diseases (HR 1.83, 95% CI [1.40, 2.40]; *p* < 0.001), cancer (HR 1.28, 95% CI [1.07, 1.53]; *p* < 0.001), other natural causes (HR 1.53, 95% CI [1.39, 1.70]; *p* < 0.001), and unnatural causes (HR 1.17, 95% CI [1.06, 1.29]; *p* < 0.001). When stratified by attained age, maternal obesity was associated with increased offspring mortality due to respiratory diseases and congenital anomalies during infancy and early childhood. In contrast, in older age groups, maternal obesity showed stronger associations with mortality from chronic conditions, including cardiovascular diseases, neurological disorders, and cancer (S5 Table).

Mediation analyses suggested that the associations of maternal obesity with offspring mortality were largely independent of hypertensive disorders of pregnancy, gestational diabetes mellitus, or inappropriate for gestational age. Preterm birth mediated 16.0% of the observed association (Table 6). In the combined mediation analysis including all measured perinatal factors as multiple mediators in the same model, the estimated mediated proportion was 25.8% for maternal obesity.

**Table 6:** Hazard ratios and 95% confidence intervals for the associations between maternal obesity and offspring all-cause mortality, mediation by adverse pregnancy outcomes.

	Preterm birth		Hypertensive disorders of pregnancy		Gestational diabetes mellitus		Large for gestational age		Small for gestational age		All perinatal factors	
	HR [95% CI]	*p* value	HR [95% CI]	*p* value	HR [95% CI]	*p* value	HR [95% CI]	*p* value	HR [95% CI]	*p* value	HR [95% CI]	*p* value
Total effect	1.40 [1.32, 1.48]	<0.001	1.39 [1.33, 1.46]	<0.001	1.42 [1.35, 1.50]	<0.001	1.41 [1.34, 1.50]	<0.001	1.40 [1.34, 1.47]	0.001	1.44 [1.37, 1.52]	<0.001
Natural direct effect	1.35 [1.27, 1.42]	<0.001	1.37 [1.30, 1.44]	<0.001	1.43 [1.26, 1.51]	<0.001	1.40 [1.33, 1.49]	<0.001	1.41 [1.34, 1.48]	<0.001	1.33 [1.26, 1.40]	<0.001
Natural indirect effect	1.04 [1.03, 1.04]	<0.001	1.02 [1.01, 1.02]	<0.001	0.99 [0.99, 1.00]	0.09	1.01 [1.00, 1.01]	0.09	1.00 [0.99, 1.00]	0.07	1.09 [1.07, 1.09]	<0.001
Proportion mediated, %	16.0		2.2		0.0		1.0		−1.0		25.8	

HR, adjusted hazard ratio; CI, confidence interval; Models were adjusted for offspring age (time since birth as the underlying time scale), calendar year of birth, sex, birth order, and maternal characteristics (age, education, and income at the childbirth, marital status, country of birth, smoking status in early pregnancy, psychiatric disorders and cardiovascular diseases before the childbirth); In analyses considering gestational diabetes mellitus as a mediator or a combined mediation analysis including all measured perinatal factors, the study population was restricted to offspring born during 1987–2014, because International Classification of Diseases codes for gestational diabetes mellitus were available only from 1987 onward. P values for the total, natural direct, and natural indirect effects were calculated using nonparametric bootstrap inference.

The results were similar in the analysis using multiple imputation for missing data (S6 Table). The associations of maternal BMI with offspring mortality remained unchanged when follow-up was restricted to 2014 (S7 Table). Associations were similar in case of neonatal and post-neonatal infant mortality (S8 Table). The associations were also largely unchanged when offspring from pregnancies affected by each perinatal factors, including preterm birth, hypertensive disorders of pregnancy, gestational diabetes mellitus, hypoxic-ischemic encephalopathy, and large or small for gestational age were excluded separately, as well as when those affected by any of these perinatal factors were excluded simultaneously (S9 Table). The association between maternal BMI and the risk of mortality in offspring did not substantially differ by maternal country of birth (S10 Table), education (S11 Table), calendar period of offspring birth (S12 Table), or birth order (S13 Table).

## Discussion

In this large population-based study, we found that maternal overweight and obesity during early pregnancy were associated with increased risks of offspring mortality. The associations (1) were stronger with increasing severity of maternal overweight and obesity, (2) persisted across infancy, adolescent, and adulthood, but was absent during childhood, (3) for specific common causes of mortality, i.e., respiratory diseases, cardiovascular diseases, congenital anomalies, and unnatural causes were consistent with findings from the primary analysis, and (4) remained directionally consistent but somewhat attenuated in cousin comparison analyses compared to those observed in the primary analysis.

To our knowledge, this is the first study to investigate the association between maternal BMI during early pregnancy and the risk of all-cause and cause-specific mortality in offspring up to early adulthood. A previous Swedish study reported that compared to offspring of mothers with normal weight, offspring of mothers with overweight and obesity grade I, II, and III had 1.25, 1.37, 2.11, and 2.44 times higher infant mortality risks, respectively [13]. The study focused only on infant mortality and did not employ a family-based design to account for unmeasured shared familial confounding [13]. A recent US nationwide cohort study found that maternal obesity, but not overweight, had a dose-dependent association with sudden unexpected infant death [14]. Only one earlier study investigated the association between maternal obesity and the risk of offspring mortality beyond infancy [15]. This Scottish cohort study followed 37,709 individuals born during 1950–1976 up to 2012 and reported, in line with our findings, that maternal obesity was associated with an increased risk of all-cause mortality in adult offspring aged 34–61 years [15]; however, their limited statistical power did not allow investigating the importance of cause-specific mortality in this association. We found that maternal overweight and obesity were associated with increased risks of mortality due to respiratory diseases, cardiovascular diseases, congenital anomalies, other natural causes, and unnatural deaths.

Our study contributes to evidence in this area in several important ways. First, with our large sample size, we were able to show a dose-response association between maternal obesity and the risk of offspring mortality, as well as to study cause-specific mortality in offspring. Second, with a follow-up of up to 42 years, we showed that the increased risks of offspring mortality associated with overweight and obesity persisted through adulthood. Third, we were the first to employ a family-based design, specifically, cousin comparison analysis, to account for unmeasured shared familial confounding. The results suggested that the observed associations could not be fully explained by shared genetic or environmental factors within families.

We observed associations between maternal early pregnancy BMI and offspring death due to congenital anomalies, respiratory diseases, cardiovascular diseases and other natural causes. These findings are in line with previous studies showing associations between maternal obesity and congenital malformations [29], asthma [30], pulmonary conditions [31], hypertension [32], cancer [33], and cerebrovascular diseases [11]. We speculate that the observed link between maternal overweight and obesity and the increased risk of unnatural deaths, including accidents, suicide, and other unnatural causes may be partly explained by the fact that maternal overweight and obesity are associated with increased risks of psychiatric disorders in offspring [34]. We observed a tendency toward increased risk of neurological disease; however, the confidence interval just included 1, suggesting that this finding should be interpreted with caution. Further studies with an extended follow-up of this cohort or analyses from other comparable large cohorts are needed to clarify the associations between maternal BMI in early pregnancy and neurological diseases.

The associations observed between maternal overweight or obesity in early pregnancy and offspring mortality are likely to be multifactorial and should be interpreted cautiously. Although several biological pathways may be involved, the findings may also reflect maternal comorbidities, shared familial susceptibility, lifestyle factors, and broader socioeconomic influences. Maternal overweight and obesity have been associated with an increased risk of adverse pregnancy outcomes, which may in turn contribute to higher risks of offspring morbidity and mortality [16,17]. The estimated proportion mediated by these perinatal factors was 25.8% for maternal obesity. In addition, the developmental origins of health and disease theory suggests that adverse intrauterine conditions may influence fetal development in ways that have long-term implications for offspring health [35]. Maternal obesity has also been linked to low-grade inflammation and metabolic disturbances during pregnancy, which may plausibly affect fetal growth and later susceptibility to diseases [36]. Altered epigenetic processes may be another potential mechanism [37]. Evidence from experimental studies suggests that obesity during pregnancy may influence offspring health through epigenetic alternations related to neurotransmitter systems, including serotonin and dopamine pathways, oxidative stress, and regulation of corticosteroid-receptor expression [38,39].

At the same time, these potential biological mechanisms should not be interpreted as evidence of a direct causal effect of maternal BMI on offspring mortality. Maternal BMI may also function as a marker of a broader set of health and social conditions that cluster within families. For example, genetic predisposition, maternal physical and mental health, health-related behaviors, and socioeconomic circumstances may all contribute to the observed associations [40,41]. In the cousin comparison analysis, the associations were attenuated, which may suggest that shared familial factors partly account for the findings. However, this design does not account for non-shared factors, such as individual dietary patterns, sedentary behaviors, or alcohol use. Taken together, the observed associations likely reflect a combination of biological, familial, behavioral, and social processes rather than a direct effect of maternal BMI. The findings for unnatural causes of death, for which the biological basis of a direct association with maternal BMI is less clear, should therefore be interpreted with particular caution, as these associations may be more likely to reflect residual confounding or unmeasured familial and social factors.

Our study had several strengths. First, the large, population-based study using nationwide registers limits the possibility of selection bias and loss to follow-up. Second, the large sample size allowed us to divide the follow-up according to period of life, i.e., childhood, adolescence, and early adulthood. Third, maternal weight during early pregnancy was measured objectively and prospectively, which could rule out recall bias for the exposure. Fourth, through linkage of several high-quality national registers, we could obtain information on several important confounders. Our study had also limitations. First, we lacked complete and harmonized information on offspring characteristics, such as BMI, socioeconomic status, and lifestyle factors (e.g., diet, smoking and alcohol behavior, or physical activity), all of which could be mediators of the association of maternal obesity and offspring premature death. Further studies are warranted to better understand the potential roles of these offspring characteristics in the observe associations. Second, maternal BMI during early pregnancy was available for 83% of all women, with missingness concentrated in the earlier years of the study period. This may have reduced the representation of earlier birth cohorts and introduced selection bias if women with recorded BMI differed systematically from those without recorded BMI in characteristics associated with offspring mortality. The results remained similar when restricting the cohort to offspring born in 1992 or later, when BMI data were more complete, and when applying multiple imputation, providing some reassurance regarding the robustness of our findings. Nevertheless, some bias due to missing BMI cannot be ruled out because multiple imputation relies on the missing at random assumption and BMI missingness varied by calendar period. Furthermore, information on the timing of the first prenatal visit was not available [18]. Although the first antenatal visit in Sweden occurs within the first trimester for approximately 90% of pregnant women, some misclassification of maternal BMI cannot be excluded. Third, although cousin comparison analyses can partially account for familial factors shared within extended families, some residual genetic and familial confounding may remain, particularly from factors that vary within extended families or across pregnancies. For example, women with a high BMI might have a less healthy lifestyle, including a poor diet or higher alcohol consumption than women who maintain a normal BMI. In addition, although we adjusted for several maternal conditions available across the study period, including maternal psychiatric disorders and cardiovascular disease, and some other pre-existing maternal disease that were less completely recorded in earlier calendar years, the possibility of residual confounding by pre-existing maternal disease cannot be completely excluded. Fourth, the prevalence of maternal obesity in Swedish women and rates of child and premature mortality are lower than in many other parts of the world [42]. Thus, the generalizability of our findings to other populations, especially to those from low- and middle-income countries, could be limited. Future studies in other populations are needed. Fifth, although we conducted additional stratified analyses by maternal country of birth, detailed information on racial or ethnic background was not available. As a result, we could not directly examine whether the association differs across racial or ethnic groups. Sixth, the mediation analysis should be interpreted cautiously, as causal mediation analysis with survival outcomes is methodological more complex than mediation analysis for standard continuous or categorical outcomes and relies on strong assumptions, including lack of unmeasured confounding.

In this large national cohort study, we found that maternal overweight and obesity are associated with increased risks of offspring mortality up to early adulthood. The associations could not be fully explained by shared familial confounding. Although our findings do not establish causality, they highlight the potential clinical and public health relevance of maternal weight before and during pregnancy and suggest that maternal BMI may help identify offspring at elevated long-term health risk. Further studies using complementary epidemiological and, where feasible, intervention studies, are needed to clarify whether the observed association reflects a causal relationship and whether maternal weight optimization may improve long-term offspring health outcomes.

## Supporting information

S1 Table Characteristics of offspring excluded from and included in the study due to missing data on maternal body mass index during early pregnancy. (DOCX)

S2 Table International Classification of Diseases codes used in the study. (DOCX)

S3 Table Characteristics of study participants and rates of mortality by offspring death. (DOCX)

S4 Table Distribution of maternal body mass index in early pregnancy by calendar year, 1982–2014, except 1990 and 1991. (DOCX)

S5 Table Associations between maternal body mass index during early pregnancy and risk of cause-specific mortality in offspring, stratified by attained age of the offspring, adjusted hazard ratio and 95% confidence intervals. (DOCX)

S6 Table Association between maternal body mass index in early pregnancy and risk of all-cause mortality in offspring using multiple imputation, adjusted hazard ratios and 95% confidence intervals. (DOCX)

S7 Table Associations between maternal body mass index in early pregnancy and risks of all-cause mortality in offspring, overall and stratified by attained age of the offspring, adjusted hazard ratios and 95% confidence intervals with follow-up until 2014. (DOCX)

S8 Table Associations between maternal body mass index in early pregnancy and risks of all-cause infant mortality in offspring, stratified by neonatal and post-neonatal period, adjusted hazard ratios and 95% confidence intervals. (DOCX)

S9 Table Association between maternal body mass index in early pregnancy and risk of all-cause mortality in offspring, excluding offspring from pregnancies affected by specific perinatal factors, adjusted hazard ratios and 95% confidence intervals. (DOCX)

S10 Table Association between maternal body mass index in early pregnancy and risks of all-cause mortality in offspring, stratified by maternal country of birth, adjusted hazard ratios and 95% confidence intervals. (DOCX)

S11 Table Association between maternal body mass index in early pregnancy and risks of all-cause mortality in offspring, stratified by maternal education, adjusted hazard ratios and 95% confidence intervals. (DOCX)

S12 Table Association between maternal body mass index in early pregnancy and risks of all-cause mortality in offspring, stratified by calendar year of offspring birth, adjusted hazard ratios and 95% confidence intervals. (DOCX)

S13 Table Association between maternal body mass index in early pregnancy and risks of all-cause mortality in offspring, stratified by birth order, adjusted hazard ratios and 95% confidence intervals. (DOCX)

S1 Fig Flowchart of the study population. (DOCX)

S2 Fig Directed Acyclic Graph for the study variables. (DOCX)

S3 Fig Log-minus-log survival plots of offspring mortality by maternal body mass index category. (DOCX)

S4 Fig Association between maternal body mass index in early pregnancy and offspring all-cause mortality, in restricted cubic spline analysis, adjusted hazard ratios and 95% confidence interval. (DOCX)

S1 STROBE Checklist Checklist for cohort studies from the STROBE Statement (https://www.strobe-statement.org/), licensed under the Creative Commons Attribution 4.0 International License (CC BY 4.0). (DOC)

S1 File Analyses plan. (DOCX)

## Abbreviations

BMI: body mass index
CI: confidence intervals
HRs: hazard ratios
ICD: International Classification of Diseases
MBR: Medical Birth Register
RECORD: Reporting of Studies Conducted using Observational Routinely-Collected Data
