# Supplementary material for: Maternal body mass index in early pregnancy and offspring mortality up to early adulthood: A nationwide cohort study
Source: PLoS Med. 2026 Sep 1;23(9):e1005217. doi: 10.1371/journal.pmed.1005217 (PMC13533357; doi:10.1371/journal.pmed.1005217)
Supplement: Supplementary file 1 [file pmed.1005217.s001.docx]

S1 Table. Characteristics of offspring excluded from and included in the study due to missing data on maternal body mass index during early pregnancy

| Characteristics | Excluded offspring | Included offspring |
| --- | --- | --- |
|  | N (%) | N (%) |
| Sex |  |  |
| Males | 275,274 (51.5) | 1,339,365 (51.4) |
| Females | 259,650 (48.5) | 1,267,319 (48.6) |
| Missing | NA | 0 (0) |
| Birth order |  |  |
| 1 | 211,966 (39.6) | 1,112,118 (42.7) |
| 2 | 193,845 (36.2) | 949,735 (36.4) |
| ≥3 | 129,115 (24.1) | 544,831 (20.9) |
| Calendar year of offspring birth |  |  |
| 1982-1985 | 114,304 (21.4) | 256,081 (9.8) |
| 1986-1989 | 129,356 (24.2) | 299,179 (11.5) |
| 1992-1995 | 84,777 (15.8) | 362,451 (13.9) |
| 1996-1999 | 54,263 (10.1) | 297,318 (11.4) |
| 2000-2003 | 52,442 (9.8) | 314,825 (12.1) |
| 2004-2007 | 50,140 (9.4) | 355,247 (13.6) |
| 2008-2011 | 29,523 (5.5) | 407,338 (15.6) |
| 2012-2014 | 20,121 (3.8) | 314,245 (12.1) |
| Maternal age (years) |  |  |
| ≤24 | 112,835 (21.1) | 486,238 (18.7) |
| 25-29 | 178,623 (33.4) | 871,777 (33.4) |
| 30-34 | 157,149 (29.4) | 812,754 (31.2) |
| ≥35 | 86,319 (16.1) | 435,915 (16.7) |
| Maternal education (years) |  |  |
| ≤9 | 92,045 (17.2) | 358,769 (13.8) |
| 10-12 | 249,635 (46.7) | 1,209,194 (46.4) |
| >12 | 172,671 (32.3) | 980,442 (37.6) |
| Missing | 20 575 (3.8) | 58 279 (2.2) |
| Maternal income |  |  |
| Lowest tertile | 183,333 (34.3) | 896,142 (34.4) |
| Middle tertile | 173,124 (32.4) | 873,741 (33.5) |
| Highest tertile | 174,376 (32.6) | 832,724 (31.9) |
| Missing | 4,093 (0.8) | 4,077 (0.2) |
| Maternal country of birth |  |  |
| Sweden | 437,750 (81.8) | 2,144,833 (82.3) |
| Other country | 96,964 (18.1) | 460,117 (17.7) |
| Missing | 212 (0.1) | 1,734 (0.1) |
| Maternal marital status |  |  |
| Married or register partnership | 268,205 (50.1) | 1,125,598 (43.2) |
| Others | 255,992 (47.9) | 1,390,714 (53.4) |
| Missing | 10,729 (2.0) | 90,372 (3.4) |
| Maternal smoking status in early pregnancy |  |  |
| No | 310,823 (58.1) | 2,169,398 (83.2) |
| Yes | 84,542 (15.8) | 363,685 (14.0) |
| Missing | 139,561 (26.1) | 73,601 (2.8) |
| Maternal psychiatric disorders before childbirth |  |  |
| No | 509,276 (95.2) | 2,437,409 (93.5) |
| Yes | 25,650 (4.8) | 169,275 (6.5) |
| Maternal cardiovascular disease before childbirth |  |  |
| No | 525,167 (98.2) | 2,541,058 (97.5) |
| Yes | 9,759 (1.8) | 65,626 (2.5) |
| Maternal pre-existing diabetes |  |  |
| No | 393,214 (73.5) | 226,4952 (86.9) |
| Yes | 1,989 (0.4) | 10,613 (0.4) |
| Missing | 139,723 (26.1) | 331,119 (12.7) |
| Maternal autoimmune disease |  |  |
| No | 126,959 (23.7) | 1,218,477 (46.7) |
| Yes | 10,151 (1.9) | 100,059 (3.8) |
| NA | 397,816 (74.4) | 1,288,148 (49.4) |
| Maternal family history of cardiovascular diseases |  |  |
| No | 337,837 (63.2) | 1,584,234 (60.8) |
| Yes | 122,432 (22.9) | 687,604 (26.4) |
| NA | 74,657 (14.0) | 334,846 (12.8) |
| Maternal family history of psychiatric disorders |  |  |
| No | 407,799 (76.2) | 1,975,054 (75.8) |
| Yes | 52,470 (9.8) | 296,784 (11.4) |
| NA | 74,657 (14.0) | 334,846 (12.8) |
| Preterm birth (<37 gestational weeks) |  |  |
| No | 487,781 (91.2) | 2,459,520 (94.4) |
| Yes | 44,210 (8.3) | 145,326 (5.6) |
| Missing | 2935 (0.5) | 1838 (0.1) |
| Hypertensive disorders of pregnancy |  |  |
| No | 516,911 (96.6) | 2,511,357 (96.3) |
| Yes | 18,015 (3.4) | 95,327 (3.7) |
| Gestational diabetes mellitus |  |  |
| No | 391,697 (73.2) | 2,255,275 (86.5) |
| Yes | 3,506 (0.7) | 20,290 (0.8) |
| NA | 139,723 (26.1) | 33,1119 (12.7) |
| Large for gestational age (>97^th^ percentile) |  |  |
| No | 507,212 (94.8) | 2,488,942 (95.5) |
| Yes | 22,374 (4.2) | 108,452 (4.2) |
| Missing | 5340 (1.0) | 9290 (0.4) |
| Small for gestational age (<3^rd^ percentile) |  |  |
| No | 507,361 (94.8) | 2,508,879 (96.2) |
| Yes | 22,225 (4.2) | 88,515 (3.4) |
| Missing | 5340 (1.0) | 9290 (0.4) |
| Hypoxic-ischemic encephalopathy |  |  |
| No | 192,023 (35.9) | 1,608,786 (61.7) |
| Yes | 68 (<0.01) | 797 (<0.01) |
| Missing | 342,835 (64.1) | 997,101 (38.3) |

NA, indicate less than 5 cases in a cell; Data on pre-existing diabetes, gestational diabetes mellitus, hypoxic-ischemic encephalopathy, and autoimmune disease are shown as missing for births before 1987, 1987, 1997, and 2001, respectively.
