# Supplementary material for: Maternal body mass index in early pregnancy and offspring mortality up to early adulthood: A nationwide cohort study
Source: PLoS Med. 2026 Sep 1;23(9):e1005217. doi: 10.1371/journal.pmed.1005217 (PMC13533357; doi:10.1371/journal.pmed.1005217)
Supplement: Supplementary file 2 [file pmed.1005217.s002.docx]

S2 Table. International Classification of Diseases codes used in the study

|  | ICD revision | | |
| --- | --- | --- | --- |
|  | ICD-8 | ICD-9 | ICD-10 |
| **Diseases**  Psychiatric disorders | 290-315 | 290-315 | F00-F99 |
| Cardiovascular diseases | 390-458 | 390-458 | I00-I99 |
| Pre-existing diabetes | NA | 250; 648A | E10-E14;  O24.0-O24.3 |
| Autoimmune disease | 099.3; 135; 136.0;269.00; 269.98;  281.0; 283.90;  283.92; 287.00;  287.3; 340; 341;  354.01; 390; 391;  392; 446; 446.20; 446.30; 446.31;  446.39; 563.00; 563.10; 569.02; 580;582; 693.99;  694.00; 694.05; 696; 704.00; 709.05; 710.0; 711.1; 712.00; 712.10; 712.20; 712.38; 712.39; 712.4; 716.00;  716.10; 717.9; 734.1; 734.90; 734.98; 734.91; 734.99 | 099D; 135; 136B;  242A; 242X;  244W; 244X;  245C; 245W;  250; 255E; 258B;  281A; 283A;  287A; 287D;  340; 341A; 357A;  358A; 390; 391;  392; 446A;  446B; 446E-G;  555; 556; 571G;  579G; 580; 582;  694A; 694E-F;  696A-B; 704A;  709A; 710A-E;  710X; 710W; 711A;  714A-D;  719D; 720A;  725 | D51.0; D59.0;  D59.1; D69.0;  D69.3; D86;  E03.5; E03.9;  E05.0; E05.5;  E05.9; E06.3;  E06.5; E10;  E27.1; E27.2;  E31.0; G04;  G13.1; G35;  G36; G61.0;  G61.1; G61.8-9;  G70; I00; I01.0-2;  I01.8-9; I02.0; I02.9; K50-51;  K74.3; K90.0;  L10.0; L12; L13.0;  L40; L63; L80;  M02.3; M02.8-9;  M05-06; M08.0-4; M30; M31.1; M31.3;  M31.5-M31.7  M32; M33.0-2;  M33.9; M35.3;  M34; M35.0;  M35.1; M35.2  N00; N01;  N03; N05 |
| Hypertensive disorders of pregnancy | 637.01; 637.03;  637.04; 637.09; 637.99; 637.10 | 642D-G;  642X | O13-16 |
| Gestational diabetes mellitus |  | 648W | O244 |
| Hypoxic-ischemic encephalopathy |  |  | P916B, P916C,  P916X |
| **Death** |  |  |  |
| Due to congenital anomalies | 740-759 | 740-759 | Q00-Q99 |
| Due to respiratory diseases | 460-519; 776 | 460-519; 768-770 | J00-J99; P20-P28 |
| Due to cardiovascular diseases | 390-458 | 390-458 | I00-I99 |
| Due to neurological diseases | 320-359 | 320-359 | G00-G99 |
| Due to cancer | 140-209 | 140-209 | C00-C99 |
| Due to unnatural causes | 796-999;  E800-999 | 798X; 800-999;  E800-999 | S00-T98;  V01-Y98 |

ICD, International Classification of Diseases; NA, not appliable.
