# Supplementary material for: Maternal body mass index in early pregnancy and offspring mortality up to early adulthood: A nationwide cohort study
Source: PLoS Med. 2026 Sep 1;23(9):e1005217. doi: 10.1371/journal.pmed.1005217 (PMC13533357; doi:10.1371/journal.pmed.1005217)
Supplement: Supplementary file 3 [file pmed.1005217.s003.docx]

S3 Table. Characteristics of study participants and rates of mortality by offspring death

| Characteristics | Offspring death | | |
| --- | --- | --- | --- |
|  | No (N=2,584,703) | Yes (N=21,981) | |
|  | Number (%) | Number (%) | Rate^*^ |
| Sex |  |  |  |
| Males | 1,325,624 (51.3) | 13,741 (62.5) | 4.4 |
| Females | 1,259,079 (48.7) | 8,240 (37.5) | 2.8 |
| Birth order |  |  |  |
| 1 | 1,103,229 (42.7) | 8,889 (40.4) | 3.5 |
| 2 | 942,173 (36.5) | 7,562 (34.4) | 3.4 |
| ≥3 | 539,301 (20.9) | 5,530 (25.2) | 4.2 |
| Calendar year of offspring birth |  |  |  |
| 1982-1985 | 251,135 (9.7) | 4,946 (22.5) | 5.0 |
| 1986-1989 | 294,484 (11.4) | 4,695 (21.4) | 4.5 |
| 1992-1995 | 358,202 (13.9) | 4,249 (19.3) | 4.0 |
| 1996-1999 | 294,861 (11.4) | 2,457 (11.2) | 3.2 |
| 2000-2003 | 312,843 (12.1) | 1,982 (9.0) | 2.9 |
| 2004-2007 | 353,745 (13.7) | 1,502 (6.8) | 2.4 |
| 2008-2011 | 406,060 (15.7) | 1,278 (5.8) | 2.3 |
| 2012-2014 | 313,373 (12.1) | 872 (4.0) | 2.7 |
| Maternal age (year) |  |  |  |
| ≤24 | 480,447 (18.6) | 5,791 (26.3) | 4.6 |
| 25-29 | 864,560 (33.4) | 7,217 (32.8) | 3.4 |
| 30-34 | 806,954 (31.2) | 5,800 (26.4) | 3.2 |
| ≥35 | 432,742 (16.7) | 3,173 (14.4) | 3.5 |
| Maternal education (year) |  |  |  |
| ≤9 | 353,932 (13.7) | 4,837 (22.0) | 5.3 |
| 10-12 | 1,198,300 (46.4) | 10,894 (49.6) | 3.6 |
| >12 | 974,778 (37.7) | 5,664 (25.8) | 2.8 |
| Missing | 57,693 (2.2) | 586 (2.7) | 5.2 |
| Maternal income |  |  |  |
| Lowest tertile | 888,184 (34.4) | 7,958 (36.2) | 3.6 |
| Middle tertile | 866,531 (33.5) | 7,210 (32.8) | 3.5 |
| Highest tertile | 825,946 (32.0) | 6,778 (30.8) | 3.8 |
| Missing | 4,042 (0.2) | 35 (0.2) | 3.0 |
| Maternal country of birth |  |  |  |
| Sweden | 2,126,560 (82.3) | 18,273 (83.1) | 3.5 |
| Other country | 456,421 (17.7) | 3,696 (16.8) | 4.1 |
| Missing | 1,722 (0.1) | 12 (0.1) | 4.5 |
| Marital status |  |  |  |
| Married or register partnership | 1,115,321 (43.2) | 10,277 (46.8) | 3.6 |
| Others | 1,379,308 (53.4) | 11,406 (51.9) | 3.7 |
| Missing | 90,074 (3.5) | 298 (1.4) | 2.4 |
| Maternal smoking status during early pregnancy | |  |  |
| No | 2,154,269 (83.3) | 15,129 (68.8) | 3.1 |
| Yes | 357,977 (13.8) | 5,708 (26.0) | 5.5 |
| Missing | 72,457 (2.8) | 1,144 (5.2) | 5.0 |
| Maternal psychiatric disorders before childbirth | | | |
| No | 2,416,906 (93.5) | 20,503 (93.3) | 3.6 |
| Yes | 167,797 (6.5) | 1,478 (6.7) | 5.0 |
| Maternal cardiovascular diseases before childbirth | | | |
| No | 2,519,529 (97.5) | 21,529 (97.9) | 3.6 |
| Yes | 65,174 (2.5) | 452 (2.1) | 3.9 |
| Maternal pre-existing diabetes |  |  |  |
| No | 2,249,326 (87.0) | 15,626 (71.1) | 3.3 |
| Yes | 10,495 (0.4) | 118 (0.5) | 5.8 |
| Missing | 324,882 (12.6) | 6,237 (28.4) | 4.9 |
| Maternal autoimmune disease before childbirth | | | |
| No | 1,213,791 (47.0) | 4,686 (21.3) | 2.5 |
| Yes | 99,629 (3.9) | 430 (2.0) | 2.9 |
| Missing | 1,271,283 (49.2) | 16,865 (76.7) | 4.2 |
| Maternal family history of cardiovascular diseases before childbirth | | | |
| No | 1,569,726 (60.7) | 14,508 (66.0) | 3.7 |
| Yes | 682,744 (26.4) | 4,860 (22.1) | 3.3 |
| Missing | 332,233 (12.9) | 2,613 (11.9) | 4.1 |
| Maternal family history of psychiatric disorders before childbirth | | | |
| No | 1,958,148 (75.8) | 16,906 (76.9) | 3.5 |
| Yes | 294,322 (11.4) | 2,462 (11.2) | 3.9 |
| Missing | 332,233 (12.9) | 2,613 (11.9) |  |
| Preterm birth (<37 gestational weeks) |  |  |  |
| No | 2,442,451 (94.5) | 17,069 (77.7) | 3.0 |
| Yes | 140,468 (5.4) | 4,858 (22.1) | 14.9 |
| Missing | 1,784 (0.1) | 54 (0.2) | 8.3 |
| Hypertensive disorders of pregnancy |  |  |  |
| No | 2,490,367 (96.4) | 20,990 (95.5) | 3.6 |
| Yes | 94,336 (3.6) | 991 (4.5) | 4.6 |
| Gestational diabetes mellitus |  |  |  |
| No | 2,239,652 (86.7) | 15,623 (71.1) | 3.3 |
| Yes | 20,169 (0.8) | 121 (0.6) | 3.1 |
| NA | 324,882 (12.6) | 6,237 (28.4) | 4.9 |
| Large for gestational age (>97^th^ percentile) | |  |  |
| No | 2,468,541 (95.5) | 20,401 (92.8) | 3.7 |
| Yes | 107,494 (4.2) | 958 (4.4) | 3.2 |
| Missing | 8,668 (0.3) | 622 (2.8) | 8.3 |
| Small for gestational age (<3^rd^ percentile) | |  |  |
| No | 2,490,089 (96.3) | 18,790 (85.5) | 3.2 |
| Yes | 85,946 (3.3) | 2,569 (11.7) | 12.5 |
| Missing | 8,668 (0.3) | 622 (2.8) | 24.1 |
| Hypoxic-ischemic encephalopathy |  |  |  |
| No | 1,601,454 (62.0) | 7,332 (33.4) | 2.7 |
| Yes | 711 (<0.1) | 86 (0.4) | 96.7 |
| Missing | 982,538 (38.0) | 14,563 (66.3) | 4.4 |

^*^Rate per 10 000 person-years; Data on pre-existing diabetes, gestational diabetes mellitus, hypoxic-ischemic encephalopathy, and autoimmune disease are shown as missing for births before 1987, 1987, 1997, and 2001, respectively.
