# Supplementary material for: Maternal body mass index in early pregnancy and offspring mortality up to early adulthood: A nationwide cohort study
Source: PLoS Med. 2026 Sep 1;23(9):e1005217. doi: 10.1371/journal.pmed.1005217 (PMC13533357; doi:10.1371/journal.pmed.1005217)
Supplement: Supplementary file 4 [file pmed.1005217.s004.docx]

S4 Table. Distribution of maternal body mass index in early pregnancy by calendar year, 1982 to 2014, except 1990 and 1991

| Year | Maternal body mass index category, kg/m^2^ | | | | | |
| --- | --- | --- | --- | --- | --- | --- |
|  | <18.5 | 18.5-24.9 | 25.0-29.9 | 30.0-34.9 | 35.0-39.9 | ≥40 |
| 1982 | 9.47 | 77.76 | 10.75 | 1.86 | 0.14 | 0.01 |
| 1983 | 8.90 | 78.07 | 10.96 | 1.90 | 0.14 | 0.03 |
| 1984 | 7.49 | 78.50 | 11.85 | 2.00 | 0.16 | 0.01 |
| 1985 | 7.27 | 77.97 | 12.35 | 2.23 | 0.16 | 0.00 |
| 1986 | 8.54 | 77.07 | 12.08 | 2.14 | 0.16 | 0.01 |
| 1987 | 8.04 | 76.78 | 12.80 | 2.23 | 0.14 | 0.00 |
| 1988 | 7.98 | 76.22 | 13.29 | 2.36 | 0.15 | 0.01 |
| 1989 | 7.85 | 76.20 | 13.49 | 2.30 | 0.15 | 0.00 |
| 1992 | 3.58 | 70.93 | 19.53 | 4.61 | 1.12 | 0.23 |
| 1993 | 3.49 | 69.88 | 20.39 | 4.89 | 1.10 | 0.24 |
| 1994 | 3.25 | 68.56 | 21.22 | 5.39 | 1.30 | 0.28 |
| 1995 | 3.05 | 67.37 | 21.92 | 5.89 | 1.48 | 0.29 |
| 1996 | 2.97 | 66.35 | 22.84 | 6.02 | 1.47 | 0.34 |
| 1997 | 2.73 | 65.52 | 23.23 | 6.48 | 1.63 | 0.40 |
| 1998 | 2.66 | 64.06 | 24.14 | 6.89 | 1.81 | 0.44 |
| 1999 | 2.56 | 63.04 | 24.78 | 7.14 | 1.97 | 0.51 |
| 2000 | 2.56 | 62.85 | 24.74 | 7.37 | 2.01 | 0.48 |
| 2001 | 2.41 | 62.10 | 25.18 | 7.57 | 2.20 | 0.55 |
| 2002 | 2.35 | 61.92 | 25.20 | 7.59 | 2.31 | 0.63 |
| 2003 | 2.36 | 62.27 | 24.65 | 7.76 | 2.31 | 0.65 |
| 2004 | 2.21 | 61.71 | 25.12 | 7.87 | 2.41 | 0.69 |
| 2005 | 2.23 | 61.73 | 25.04 | 7.84 | 2.51 | 0.65 |
| 2006 | 2.44 | 61.43 | 24.83 | 8.04 | 2.51 | 0.75 |
| 2007 | 2.30 | 61.19 | 24.86 | 8.27 | 2.59 | 0.78 |
| 2008 | 2.48 | 61.18 | 24.82 | 8.19 | 2.52 | 0.80 |
| 2009 | 2.40 | 60.83 | 25.10 | 8.34 | 2.58 | 0.76 |
| 2010 | 2.45 | 60.16 | 25.01 | 8.86 | 2.77 | 0.75 |
| 2011 | 2.45 | 59.75 | 25.36 | 8.94 | 2.73 | 0.78 |
| 2012 | 2.48 | 59.22 | 25.46 | 9.14 | 2.85 | 0.85 |
| 2013 | 2.57 | 59.10 | 25.65 | 9.13 | 2.78 | 0.76 |
| 2014 | 2.71 | 59.22 | 25.19 | 9.25 | 2.86 | 0.78 |
