# Supplementary material for: Maternal body mass index in early pregnancy and offspring mortality up to early adulthood: A nationwide cohort study
Source: PLoS Med. 2026 Sep 1;23(9):e1005217. doi: 10.1371/journal.pmed.1005217 (PMC13533357; doi:10.1371/journal.pmed.1005217)
Supplement: Supplementary file 5 [file pmed.1005217.s005.docx]

S5 Table. Associations between maternal body mass index during early pregnancy and risk of cause-specific mortality in offspring, stratified by attained age of the offspring, adjusted hazard ratio and 95% confidence intervals

| Maternal | ≤1 years | | | >1 to ≤5 years | | >5 to ≤18 years | | >18 years | |
| --- | --- | --- | --- | --- | --- | --- | --- | --- | --- |
| BMI | Cases (Rate^*^) | | HR (95% CI) | Cases (Rate^*^) | HR (95% CI) | Cases (Rate^*^) | HR (95% CI) | Cases (Rate^*^) | HR (95% CI) |
| Death due to congenital anomalies | | | | | | | | | |
| <18.5 | 128 (0.47) | 1.04 (0.86, 1.26) | | 16 (0.06) | 1.11 (0.64, 1.92) | 6 (0.02) | 0.77 (0.34, 1.75) | 8 (0.03) | 0.81 (0.35, 1.86) |
| 18.5-24.9 | 1864 (0.45) | 1.00 (ref) | | 194 (0.05) | 1.00 (ref) | 145 (0.03) | 1.00 (ref) | 100 (0.03) | 1.00 (ref) |
| 25.0-29.9 | 632 (0.53) | 1.14 (1.03, 1.25) | | 75 (0.06) | 1.27 (0.95, 1.70) | 55 (0.05) | 1.36 (0.98, 1.88) | 30 (0.03) | 1.40 (0.91, 2.14) |
| ≥30 | 281 (0.65) | 1.34 (1.17, 1.54) | | 20 (0.05) | 0.91 (0.55, 1.51) | 21 (0.05) | 1.27 (0.77, 2.10) | 11 (0.04) | 1.55 (0.77, 3.12) |
| Death due to respiratory diseases | | | | | | | | | |
| <18.5 | 46 (0.17) | 0.95 (0.69, 1.30) | | NA | NA | 9 (0.03) | 2.76 (1.33, 5.70) | 9 (0.04) | 1.29 (0.64, 2.58) |
| 18.5-24.9 | 756 (0.18) | 1.00 (ref) | | 80 (0.02) | 1.00 (ref) | 47 (0.01) | 1.00 (ref) | 79 (0.02) | 1.00 (ref) |
| 25.0-29.9 | 341 (0.28) | 1.55 (1.35, 1.77) | | 30 (0.03) | 1.28 (0.82, 2.02) | 15 (0.01) | 1.09 (0.59, 2.00) | 24 (0.03) | 1.55 (0.97, 2.50) |
| ≥30 | 176 (0.40) | 2.14 (1.79, 2.56) | | 14 (0.03) | 1.53 (0.82, 2.88) | 9 (0.02) | 1.55 (0.68, 3.49) | 5 (0.02) | 1.31 (0.52, 3.28) |
| Death due to neurological diseases | | | | | | | | | |
| <18.5 | 12 (0.04) | 1.20 (0.63, 2.29) | | 5 (0.02) | 0.64 (0.23, 1.74) | 20 (0.07) | 2.17 (1.32, 3.59) | 16 (0.07) | 0.81 (0.47, 1.39) |
| 18.5-24.9 | 152 (0.04) | 1.00 (ref) | | 108 (0.03) | 1.00 (ref) | 142 (0.03) | 1.00 (ref) | 227 (0.07) | 1.00 (ref) |
| 25.0-29.9 | 61 (0.05) | 1.30 (0.95, 1.79) | | 38 (0.03) | 1.03 (0.70, 1.51) | 55 (0.05) | 1.13 (0.81, 1.59) | 54 (0.06) | 1.01 (0.74, 1.39) |
| ≥30 | 20 (0.05) | 1.08 (0.65, 1.79) | | 10 (0.02) | 0.55 (0.26, 1.14) | 30 (0.07) | 1.60 (1.04, 2.46) | 27 (0.09) | 1.82 (1.18, 2.80) |
| Death due to cardiovascular diseases | | | | | | | | | |
| <18.5 | 5 (0.02) | 1.07 (0.43, 2.68) | | 5 (0.02) | 1.49 (0.53, 4.23) | NA | NA | 25 (0.10) | 0.99 (0.65, 1.52) |
| 18.5-24.9 | 78 (0.02) | 1.00 (ref) | | 41 (0.01) | 1.00 (ref) | 102 (0.02) | 1.00 (ref) | 280 (0.08) | 1.00 (ref) |
| 25.0-29.9 | 21 (0.02) | 0.76 (0.44, 1.31) | | 14 (0.01) | 1.16 (0.61, 2.21) | 44 (0.04) | 1.50 (1.03, 2.18) | 71 (0.08) | 1.24 (0.94, 1.63) |
| ≥30 | 16 (0.04) | 1.74 (0.96, 3.14) | | 8 (0.02) | 1.90 (0.86, 4.21) | 9 (0.02) | 0.73 (0.33, 1.58) | 40 (0.14) | 2.58 (1.80, 3.69) |
| Death due to cancer | | | | | | | | | |
| <18.5 | 4 (0.01) | 1.15 (0.35, 3.76) | | 10 (0.04) | 0.58 (0.29, 1.19) | 22 (0.08) | 0.70 (0.44, 1.10) | 68 (0.28) | 1.33 (0.88, 1.47) |
| 18.5-24.9 | 46 (0.01) | 1.00 (ref) | | 241 (0.06) | 1.00 (ref) | 469 (0.11) | 1.00 (ref) | 696 (0.20) | 1.00 (ref) |
| 25.0-29.9 | 16 (0.01) | 1.19 (0.65, 2.18) | | 71 (0.06) | 0.89 (0.67, 1.19) | 145 (0.12) | 1.03 (0.85, 1.26) | 135 (0.15) | 0.93 (0.77, 1.14) |
| ≥30 | NA | NA | | 27 (0.06) | 0.93 (0.61, 1.43) | 62 (0.14) | 1.21 (0.91, 1.61) | 59 (0.20) | 1.73 (1.32, 2.28) |
| Death due to other natural causes | | | | | | | | | |
| <18.5 | 125 (0.46) | 1.17 (0.97, 1.42) | | 15 (0.05) | 0.88 (0.52, 1.48) | 26 (0.09) | 1.40 (0.91, 2.16) | 64 (0.26) | 1.03 (0.78, 1.35) |
| 18.5-24.9 | 1715 (0.41) | 1.00 (ref) | | 301 (0.07) | 1.00 (ref) | 285 (0.07) | 1.00 (ref) | 664 (0.19) | 1.00 (ref) |
| 25.0-29.9 | 680 (0.57) | 1.22 (1.11, 1.34) | | 91 (0.08) | 0.85 (0.66, 1.10) | 78 (0.06) | 0.88 (0.67, 1.15) | 140 (0.16) | 0.93 (0.77, 1.13) |
| ≥30 | 383 (0.88) | 1.64 (1.45, 1.85) | | 43 (0.10) | 1.08 (0.77, 1.52) | 49 (0.11) | 1.48 (1.06, 2.05) | 60 (0.21) | 1.53 (1.16, 2.01) |
| Death due to unnatural causes | | | | | | | | | |
| <18.5 | 35 (0.13) | 0.96 (0.65, 1.40) | | 12 (0.04) | 0.52 (0.27, 1.02) | 76 (0.28) | 1.24 (0.97, 1.60) | 391 (1.60) | 0.95 (0.85, 1.06) |
| 18.5-24.9 | 390 (0.09) | 1.00 (ref) | | 264 (0.06) | 1.00 (ref) | 815 (0.20) | 1.00 (ref) | 4276 (1.25) | 1.00 (ref) |
| 25.0-29.9 | 114 (0.09) | 1.05 (0.84, 1.31) | | 76 (0.06) | 0.88 (0.67, 1.17) | 263 (0.22) | 1.12 (0.97, 1.30) | 999 (1.11) | 1.03 (0.96, 1.11) |
| ≥30 | 35 (0.08) | 1.02 (0.72, 1.45) | | 42 (0.10) | 1.42 (1.00, 2.02) | 100 (0.23) | 1.17 (0.93, 1.46) | 331 (1.15) | 1.17 (1.04, 1.32) |

BMI, body mass index; HR, adjusted hazard ratio; CI, confidence interval; NA, indicate less than 5 cases in a cell; ^*^Rate per 10 000 person-years; Models were adjusted for offspring age (time since birth as the underlying time scale), calendar year of birth, sex, birth order, and maternal characteristics (age, education, and income at the child’s birth, marital status, country of birth, smoking status in early pregnancy, psychiatric disorders and cardiovascular diseases before the childbirth).
