# Supplementary material for: Maternal body mass index in early pregnancy and offspring mortality up to early adulthood: A nationwide cohort study
Source: PLoS Med. 2026 Sep 1;23(9):e1005217. doi: 10.1371/journal.pmed.1005217 (PMC13533357; doi:10.1371/journal.pmed.1005217)
Supplement: Supplementary file 6 [file pmed.1005217.s006.docx]

S6 Table. Association between maternal body mass index in early pregnancy and risk of all-cause mortality in offspring using multiple imputation, adjusted hazard ratios and 95% confidence intervals

| Maternal body mass index, kg/m^2^ | Number | Number of cases | Rate^*^ | HR (95% CI) |
| --- | --- | --- | --- | --- |
| <18.5 | 126,662 | 1,579 | 4.4 | 0.98 (0.92, 1.04) |
| 18.5-24.9 | 2,089,787 | 20,559 | 3.9 | 1.00 (ref) |
| 25.0-29.9 | 664,831 | 6,149 | 4.2 | 1.14 (1.10, 1.18) |
| 30.0-34.9 | 193,525 | 1,892 | 4.8 | 1.32 (1.25, 1.39) |
| 35.0-39.9 | 52,828 | 548 | 5.5 | 1.63 (1.47, 1.79) |
| 40 | 13,977 | 155 | 6.1 | 1.80 (1.49, 2.17) |

^*^Rate per 10 000 person years; HR, hazard ratio, CI, confidence intervals; Models were adjusted for offspring age (time since birth as the underlying time scale), calendar year of birth, sex, birth order, and maternal characteristics (age, education, and income at the child’s birth, marital status, country of birth, smoking status in early pregnancy, psychiatric disorders and cardiovascular diseases before the childbirth).
