# Supplementary material for: Maternal body mass index in early pregnancy and offspring mortality up to early adulthood: A nationwide cohort study
Source: PLoS Med. 2026 Sep 1;23(9):e1005217. doi: 10.1371/journal.pmed.1005217 (PMC13533357; doi:10.1371/journal.pmed.1005217)
Supplement: Supplementary file 8 [file pmed.1005217.s008.docx]

S8 Table. Associations between maternal body mass index in early pregnancy and risks of all-cause infant mortality in offspring, stratified by neonatal and post-neonatal period, adjusted hazard ratios and 95% confidence intervals

| Maternal BMI, kg/m^2^ | Number | Case (Rate^*^) | HR (95% CI) |
| --- | --- | --- | --- |
| BMI category |  |  |  |
| Attained age ≤28 days |  |  |  |
| <18.5 | 99,302 | 163 (0.6) | 1.06 (0.90, 1.26) |
| 18.5-24.9 | 1,719,560 | 2,476 (0.6) | 1.00 (ref) |
| 25.0-29.9 | 563,076 | 991 (0.8) | 1.29 (1.19, 1.39) |
| 30.0-34.9 | 166,462 | 339 (1.0) | 1.51 (1.33, 1.70) |
| 35.0-39.9 | 46,009 | 130 (1.5) | 2.21 (1.83, 2.67) |
| ≥40 | 12,275 | 32 (1.5) | 2.06 (1.41, 2.99) |
| Attained age >28 days to ≤1 years | |  |  |
| <18.5 | 99,138 | 192 (0.7) | 1.07 (0.91, 1.25) |
| 18.5-24.9 | 1,717,068 | 2,525 (0.6) | 1.00 (ref) |
| 25.0-29.9 | 562,076 | 874 (0.7) | 1.15 (1.06, 1.25) |
| 30.0-34.9 | 166,122 | 267 (0.8) | 1.18 (1.03, 1.36) |
| 35.0-39.9 | 45,878 | 107 (1.3) | 1.92 (1.57, 2.36) |
| ≥40 | 12,243 | 39 (1.8) | 2.85 (2.06, 3.93) |

HR, adjusted hazard ratio; CI, confidence interval; BMI, body mass index; ^*^Rate per 10 000 person-years; Models were adjusted for offspring age (time since birth as the underlying time scale), calendar year of birth, sex, birth order, and maternal characteristics (age, education, and income at the child’s birth, marital status, country of birth, smoking status in early pregnancy, psychiatric disorders and cardiovascular diseases before the childbirth).
