# Supplementary material for: Maternal body mass index in early pregnancy and offspring mortality up to early adulthood: A nationwide cohort study
Source: PLoS Med. 2026 Sep 1;23(9):e1005217. doi: 10.1371/journal.pmed.1005217 (PMC13533357; doi:10.1371/journal.pmed.1005217)
Supplement: Supplementary file 9 [file pmed.1005217.s009.docx]

S9 Table. Association between maternal body mass index in early pregnancy and risk of all-cause mortality in offspring, excluding offspring from pregnancies affected by specific perinatal factors, adjusted hazard ratios and 95% confidence intervals

| Maternal BMI,  Kg/m^2^ | Hazard ratio (95% confidence interval) | | | | | | | |
| --- | --- | --- | --- | --- | --- | --- | --- | --- |
|  | Excluding  preterm birth | | Excluding  hypertensive  disorders of  pregnancy | Excluding  gestational diabetes  mellitus | Excluding hypoxic-  ischemic  encephalopathy | Excluding large for  gestational age  (>97^th^ percentile) | Excluding small for gestational age  (<3^rd^ percentile) | Excluding all  birth affected by  these perinatal  factors |
|  | N=2,459,520 | | N=2,511,357 | N=2,255,275 | N = 1,608,786 | N=2,488,942 | N=2,508,879 | N=1,354,160 |
| Body mass index category | | | | | | | | |
| <18.5 | | 0.99 (0.92, 1.06) | 1.01 (0.95, 1.08) | 1.04 (0.95, 1.13) | 1.03 (0.88, 1.21) | 1.02 (0.95, 1.09) | 1.00 (0.93, 1.07) | 1.06 (0.86, 1.30) |
| 18.5-24.9 | | 1.00 (ref) | 1.00 (ref) | 1.00 (ref) | 1.00 (ref) | 1.00 (ref) | 1.00 (ref) | 1.00 (ref) |
| 25.0-29.9 | | 1.09 (1.05, 1.14) | 1.10 (1.06, 1.14) | 1.14 (1.09, 1.19) | 1.18 (1.12, 1.25) | 1.10 (1.06, 1.14) | 1.10 (1.05, 1.14) | 1.12 (1.04, 1.22) |
| 30.0-34.9 | | 1.26 (1.17, 1.35) | 1.28 (1.20, 1.36) | 1.30 (1.22, 1.39) | 1.32 (1.21, 1.43) | 1.27 (1.19, 1.35) | 1.28 (1.20, 1.37) | 1.26 (1.12, 1.42) |
| 35.0-39.9 | | 1.58 (1.39, 1.79) | 1.66 (1.49, 1.85) | 1.74 (1.56, 1.93) | 1.77 (1.56, 2.01) | 1.73 (1.56, 1.93) | 1.66 (1.48, 1.86) | 1.85 (1.54, 2.21) |
| ≥40 | | 1.81 (1.43, 2.30) | 1.87 (1.53, 2.30) | 1.96 (1.61, 2.39) | 2.02 (1.63, 2.52) | 1.91 (1.55, 2.36) | 1.95 (1.58, 2.40) | 2.11 (1.53, 2.90) |

Models were adjusted for offspring age (time since birth as the underlying time scale), calendar year of birth, sex, birth order, and maternal characteristics (age, education, and income at the child’s birth, marital status, country of birth, smoking status in early pregnancy, psychiatric disorders and cardiovascular diseases before the childbirth). In analyses excluding gestational diabetes mellitus and hypoxic-ischemic encephalopathy, the study population was restricted to offspring born during 1987–2014 and 1997–2014, respectively, because International Classification of Diseases codes for these conditions were available only from 1987 and 1997 onward, respectively.
