# Supplementary material for: Maternal body mass index in early pregnancy and offspring mortality up to early adulthood: A nationwide cohort study
Source: PLoS Med. 2026 Sep 1;23(9):e1005217. doi: 10.1371/journal.pmed.1005217 (PMC13533357; doi:10.1371/journal.pmed.1005217)
Supplement: Supplementary file 11 [file pmed.1005217.s011.docx]

S11 Table. Association between maternal body mass index in early pregnancy and risks of all-cause mortality in offspring, stratified by maternal education, adjusted hazard ratios and 95% confidence intervals

| Maternal body mass index, kg/m^2^ | Hazard ratio (95% confidence interval) | |
| --- | --- | --- |
|  | education ≤12 years | education >12 years |
|  | N=1,567,963 | N=980,442 |
| <18.5 | 1.05 (0.98, 1.13) | 0.91 (0.79, 1.06) |
| 18.5-24.9 | 1.00 (ref) | 1.00 (ref) |
| 25.0-29.9 | 1.12 (1.07, 1.16) | 1.10 (1.02, 1.18) |
| 30.0-34.9 | 1.29 (1.21, 1.38) | 1.25 (1.09, 1.44) |
| 35.0-39.9 | 1.67 (1.49, 1.87) | 1.83 (1.43, 2.32) |
| ≥40 | 1.92 (1.56, 2.36) | 2.11 (1.33, 3.35) |

HR, hazard ratio; CI, confidence intervals; Models were adjusted for offspring age (time since birth as the underlying time scale), calendar year of birth, sex, birth order, and maternal characteristics (age, income at the child’s birth, marital status, country of birth, smoking status in early pregnancy, psychiatric disorders and cardiovascular diseases before the childbirth).
