# Supplementary material for: Maternal body mass index in early pregnancy and offspring mortality up to early adulthood: A nationwide cohort study
Source: PLoS Med. 2026 Sep 1;23(9):e1005217. doi: 10.1371/journal.pmed.1005217 (PMC13533357; doi:10.1371/journal.pmed.1005217)
Supplement: Supplementary file 12 [file pmed.1005217.s012.docx]

S12 Table. Association between maternal body mass index in early pregnancy and risks of all-cause mortality in offspring, stratified by calendar year of offspring birth, adjusted hazard ratios and 95% confidence intervals

| Maternal body mass index, | Hazard ratio (95% confidence interval) | | | |
| --- | --- | --- | --- | --- |
| kg/m^2^ | 1982-1989 | 1992-1999 | 2000-2006 | 2007-2014 |
|  | N=555,260 | N=659,769 | N=670,072 | N=721,583 |
| <18.5 | 1.00 (0.92, 1.08) | 1.11 (0.97, 1.28) | 1.01 (0.80, 1.27) | 1.11 (0.97, 1.28) |
| 18.5-24.9 | 1.00 (ref) | 1.00 (ref) | 1.00 (ref) | 1.00 (ref) |
| 25.0-29.9 | 1.00 (0.94, 1.06) | 1.16 (1.09, 1.23) | 1.14 (1.05, 1.24) | 1.16 (1.09, 1.23) |
| 30.0-34.9 | 1.28 (1.13, 1.45) | 1.25 (1.13, 1.38) | 1.36 (1.20, 1.53) | 1.25 (1.13, 1.38) |
| 35.0-39.9 | 1.58 (1.04, 2.40) | 1.58 (1.34, 1.87) | 1.91 (1.61, 2.28) | 1.58 (1.34, 1.87) |
| ≥40 | 1.49 (0.21, 10.6) | 2.11 (1.56, 2.85) | 1.86 (1.35, 2.57) | 2.11 (1.56, 2.85) |

Models were adjusted for offspring age (time since birth as the underlying time scale), sex, birth order, and maternal characteristics (age, education, and income at the child’s birth, marital status, country of birth, smoking status in early pregnancy, psychiatric disorders and cardiovascular diseases before the childbirth).
