# Supplementary material for: Maternal body mass index in early pregnancy and offspring mortality up to early adulthood: A nationwide cohort study
Source: PLoS Med. 2026 Sep 1;23(9):e1005217. doi: 10.1371/journal.pmed.1005217 (PMC13533357; doi:10.1371/journal.pmed.1005217)
Supplement: Supplementary file 13 [file pmed.1005217.s013.docx]

S13 Table. Association between maternal body mass index in early pregnancy and risks of all-cause mortality in offspring, stratified by birth order, adjusted hazard ratios and 95% confidence intervals

| Maternal body mass index, | Hazard ratio (95% confidence interval) | | |
| --- | --- | --- | --- |
| kg/m^2^ | Birth order =1 | Birth order =2 | Birth order ≥3 |
|  | N=1,112,118 | N=949,735 | N=544,831 |
| <18.5 | 0.99 (0.90, 1.08) | 1.07 (0.96, 1.19) | 1.00 (0.86, 1.15) |
| 18.5-24.9 | 1.00 (ref) | 1.00 (ref) | 1.00 (ref) |
| 25.0-29.9 | 1.12 (1.06, 1.19) | 1.09 (1.02, 1.16) | 1.12 (1.04, 1.19) |
| 30.0-34.9 | 1.34 (1.20, 1.48) | 1.28 (1.16, 1.42) | 1.21 (1.09, 1.35) |
| 35.0-39.9 | 1.89 (1.59, 2.25) | 1.51 (1.26, 1.82) | 1.64 (1.38, 1.96) |
| ≥40 | 2.29 (1.68, 3.13) | 1.89 (1.35, 2.64) | 1.62 (1.15, 2.29) |

Models were adjusted for offspring age (time since birth as the underlying time scale), calendar year of birth, sex, and maternal characteristics (age, education, and income at the child’s birth, marital status, country of birth, smoking status in early pregnancy, psychiatric disorders and cardiovascular diseases before the childbirth).
