# Supplementary material for: Maternal body mass index in early pregnancy and offspring mortality up to early adulthood: A nationwide cohort study
Source: PLoS Med. 2026 Sep 1;23(9):e1005217. doi: 10.1371/journal.pmed.1005217 (PMC13533357; doi:10.1371/journal.pmed.1005217)
Supplement: Supplementary file 17 [file pmed.1005217.s017.docx]

S4 Fig. Association between maternal body mass index in early pregnancy and offspring all-cause mortality, in restricted cubic spline analysis, adjusted hazard ratios and 95% confidence interval

Models were adjusted for offspring age (time since birth as the underlying time scale), calendar year of birth, sex, birth order, and maternal characteristics (age, education, and income at the child’s birth, country of birth, smoking status in early pregnancy, psychiatric disorders and cardiovascular diseases before the childbirth).
