# Supplementary material for: Maternal body mass index in early pregnancy and offspring mortality up to early adulthood: A nationwide cohort study
Source: PLoS Med. 2026 Sep 1;23(9):e1005217. doi: 10.1371/journal.pmed.1005217 (PMC13533357; doi:10.1371/journal.pmed.1005217)
Supplement: Supplementary file 18 [file pmed.1005217.s019.docx]

Analysis plan-- Maternal body mass index in early pregnancy and

offspring mortality up to early-adulthood

Hui Wang, PhD; Jette Möller, PhD; Yajun Liang, MD, PhD; Krisztina D. László, PhD

Created at March 30, 2025

**Objective:**

To study the association between maternal body mass index (BMI) during early pregnancy and all-cause and cause-specific mortality in their offsprings.

**Study population:**

We will use data from the Swedish Medical Birth Register (MBR), which has included information on more than 98% of births in Sweden since 1973. Information on maternal weight and height measured at the first antenatal visit has been available in the MBR since 1982, except for the years 1990 and 1991. We will therefore include live births recorded in the MBR from January 1, 1982, through December 31, 2014, while excluding births occurring in 1990 and 1991.

We will exclude births with missing maternal BMI in early pregnancy and births with implausible BMI values, defined as values more than 5 standard deviations above or below the mean. The final analytic cohort will include offspring with valid information on maternal BMI in early pregnancy. We will compare basic characteristics of included and excluded births to assess potential selection related to missing maternal BMI.

Follow-up will start on the date of birth and will continue until death, emigration, or December 31, 2023, whichever occurs first. Information on emigration will be available through December 31, 2014. Using the unique personal identify numbers assigned to Swedish residents, we will link mother-child records from the MBR to the Swedish National Patient Register, Total Population Register, Education Register, Multigeneration Register, and Cause of Death Register.

**Exposure:**

Information on maternal height and weight in early pregnancy will be obtained from the Swedish Medical Birth Register. Maternal height will be based on self-reported information, and maternal weight will be based on weight measured in light clothing at the first antenatal visit. In Sweden, the first antenatal visit generally occurs during the first trimester, within the first 14 weeks of gestation for approximately 90% of pregnant women. Early-pregnancy maternal BMI will be calculated as weight in kilograms divided by height in meters squared (kg/m²). Maternal BMI will be categorized according to the World Health Organization classification as underweight (<18.5 kg/m²), normal weight (18.5–24.9 kg/m²), overweight (25.0–29.9 kg/m²), obesity grade I (30.0–34.9 kg/m²), obesity grade II (35.0–39.9 kg/m²), and obesity grade III (≥40.0 kg/m²).

**Outcome:**

The primary outcome will be all-cause mortality in offspring. Information on the date and underlying cause of death will be obtained from the Swedish Cause of Death Register. Cause-specific mortality will be examined according to the underlying cause of death. We will categorize deaths into the most common specific causes in this population, including congenital anomalies, respiratory diseases, neurological diseases, cardiovascular diseases, cancer, and unnatural causes, including suicide and accidents, according to The International Classification of Diseases codes.

**Covariates:**

We will include covariates that have been identified in previous studies as potential risk factors for both maternal obesity and offspring mortality. These will include maternal sociodemographic characteristics, maternal health conditions before the index birth, pregnancy-related factors, and offspring characteristics.

Maternal characteristics will include age at childbirth, smoking status in early pregnancy, highest attained education, country of birth, income, and history of psychiatric disorders and cardiovascular diseases before the index birth. Information on maternal age and smoking status will be obtained from the Swedish Medical Birth Register. Information on education, country of birth, and income will be obtained from the Education Register, Total Population Register, and Register of Incomes and Taxes, respectively. Information on pre-existing maternal psychiatric disorders and cardiovascular diseases will be obtained from the Swedish National Patient Register. If information on maternal education or income is unavailable for the corresponding year, we will use information from the closest available year within the five years before study entry. We will also include birth order, calendar year of offspring birth, and offspring sex, obtained from the Swedish Medical Birth Register.

Adverse pregnancy outcomes will include preterm birth, hypertensive disorders of pregnancy, gestational diabetes mellitus, and large for gestational age. Information on hypertensive disorders of pregnancy and gestational diabetes mellitus will be obtained from the Swedish Medical Birth Register using relevant International Classification of Diseases codes. Gestational age and birth weight will be obtained from the Swedish Medical Birth Register and will be used to define preterm birth and large for gestational age.

**Statistical analyses:**

We will use Cox proportional hazards regression to estimate hazard ratios (HRs) and 95% confidence intervals (CIs) for the associations between maternal BMI categories and all-cause mortality in offspring. Offspring of mothers with normal weight will be used as the reference group. The exposure categories will include maternal underweight, overweight, obesity grade I, obesity grade II, and obesity grade III, defined according to World Health Organization BMI categories. Models will be adjusted for the covariates described above. Robust sandwich variance estimators will be used to account for clustering of siblings born to the same mother.

We will assess linear trends across maternal BMI categories by including an ordinal variable for BMI category as a continuous term in the model. To examine whether the associations vary across offspring age, we will split follow-up time by attained age into developmental periods, including infancy, childhood, adolescence, and adulthood. We will also assess potential nonlinear associations between maternal BMI and offspring mortality using restricted cubic spline models, with knots placed at selected percentiles of the BMI distribution.

For cause-specific mortality, we will estimate associations between maternal BMI and deaths due to major underlying causes, including congenital anomalies, respiratory diseases, neurological diseases, cardiovascular diseases, cancer, and unnatural causes. Deaths from causes other than the cause under study will be treated as competing events, and individuals will be censored at the date of death from other causes. To ensure adequate statistical power for cause-specific mortality analyses, obesity grades I, II, and III may be combined into a single obesity category.

To address potential confounding by unmeasured familial factors shared within extended families, we will conduct maternal parallel cousin comparison analyses. Stratified Cox regression models will be used, with cousin sets treated as strata and adjustment for the same covariates as in the primary analysis. To explore whether adverse pregnancy outcomes may mediate the association between maternal BMI and offspring mortality, we will perform mediation analyses under the counterfactual framework.

**Sensitivity analyses:**

We will conduct several sensitivity and subgroup analyses to assess the robustness of the findings. First, because some offspring are expected to have missing information on maternal BMI and covariates, we will use multiple imputation by chained equations to impute missing values for maternal BMI and covariates included in the primary analyses. We will create 20 imputed datasets, fit Cox regression models separately within each dataset, and combine estimates using Rubin’s rules. Second, because the study period spans several decades during which the prevalence of maternal obesity and offspring mortality rates may have changed, we will perform analyses stratified by calendar period of offspring birth, in addition to adjusting for birth year in the main models. We will also conduct stratified analyses by maternal education and parity to assess potential effect modification.

**Software**

All statistical analyses will be performed using R version 4.4.1 or later.
